# Novel Small Molecule Hsp90/Cdc37 Interface Inhibitors Indirectly Target K-Ras-Signaling

**DOI:** 10.3390/cancers13040927

**Published:** 2021-02-23

**Authors:** Farid Ahmad Siddiqui, Hanna Parkkola, Vladimir Vukic, Christina Oetken-Lindholm, Alok Jaiswal, Alexandros Kiriazis, Karolina Pavic, Tero Aittokallio, Tiina A. Salminen, Daniel Abankwa

**Affiliations:** 1 Turku Bioscience Centre, University of Turku and Åbo Akademi University, 20520 Turku, Finland; farid.siddiqui@abo.fi (F.A.S.); hkpark@utu.fi (H.P.); vukicv@uns.ac.rs (V.V.); Christina.Oetken@abo.fi (C.O.-L.); alexandros.kiriazis@helsinki.fi (A.K.); 2 Faculty of Technology, University of Novi Sad, 21000 Novi Sad, Serbia; 3 Institute for Molecular Medicine Finland (FIMM), University of Helsinki, 00014 Helsinki, Finland; the.alok.jaiswal@gmail.com (A.J.); tero.aittokallio@helsinki.fi (T.A.); 4 Cancer Cell Biology and Drug Discovery Group, Department of Life Sciences and Medicine, University of Luxembourg, 4362 Esch-sur-Alzette, Luxembourg; karolina.pavic@uni.lu; 5 Department of Cancer Genetics, Institute for Cancer Research, Oslo University Hospital, N-0310 Oslo, Norway; 6 Centre for Biostatistics and Epidemiology (OCBE), Faculty of Medicine, University of Oslo, N-0372 Oslo, Norway; 7 Structural Bioinformatics Laboratory, Biochemistry, Faculty of Science and Engineering, Åbo Akademi University, 20520 Turku, Finland; tiina.salminen@abo.fi

**Keywords:** K-Ras, Hsp90, Cdc37, nanoclustering, cancer, drug development

## Abstract

**Simple Summary:**

The correct folding of proteins is essential for their activity. Therefore, cells have evolved protein-folding chaperones, such as Hsp90. Interestingly, in several cancer cells, Hsp90 appears to have a role that is more important than normal. The current working model suggests that, with the help of its co-chaperone, Cdc37, it stabilizes mutant kinases. However, Hsp90, together with Cdc37, assists additional proteins that may be relevant in cancer. We demonstrate that the Hsp90-dependent stability of the transcription factor HIF-1α and one of its downstream transcriptional targets, galectin-3, is important to maintain the elevated activity of the major oncogene KRAS. This is because galectin-3 stabilizes the MAPK-signaling complexes of K-Ras, which is called a nanocluster. In addition, we identified six drug-like small molecules that inhibit the Hsp90/Cdc37 protein interface at low micro molar concentrations. Given the co-occurrence of mutant KRAS with high HIF-1α and high galectin-3 levels in pancreatic cancer, our results suggest an application of Hsp90 inhibitors in this cancer type.

**Abstract:**

The ATP-competitive inhibitors of Hsp90 have been tested predominantly in kinase addicted cancers; however, they have had limited success. A mechanistic connection between Hsp90 and oncogenic K-Ras is not known. Here, we show that K-Ras selectivity is enabled by the loss of the K-Ras membrane nanocluster modulator galectin-3 downstream of the Hsp90 client HIF-1α. This mechanism suggests a higher drug sensitivity in the context of KRAS mutant, HIF-1α-high and/or Gal3-high cancer cells, such as those found, in particular, in pancreatic adenocarcinoma. The low toxicity of conglobatin further indicates a beneficial on-target toxicity profile for Hsp90/Cdc37 interface inhibitors. We therefore computationally screened >7 M compounds, and identified four novel small molecules with activities of 4 μM–44 μM in vitro. All of the compounds were K-Ras selective, and potently decreased the Hsp90 client protein levels without inducing the heat shock response. Moreover, they all inhibited the 2D proliferation of breast, pancreatic, and lung cancer cell lines. The most active compounds from each scaffold, furthermore, significantly blocked 3D spheroids and the growth of K-Ras-dependent microtumors. We foresee new opportunities for improved Hsp90/Cdc37 interface inhibitors in cancer and other aging-associated diseases.

## 1. Introduction

The 90 kDa heat shock protein, Hsp90, is an abundant protein folding chaperone for more than 400 clients, including kinases (e.g., Raf), transcription factors (e.g., HIF-1α), E3 ligases (e.g., cullin 3) and steroid hormone receptors (e.g., estrogen receptor) [1,2]. Two cytoplasmic isoforms (alpha and beta) and two paralogs that localize to the endoplasmic reticulum (Grp94) and mitochondria (TRAP1) are known [3]. All of these Hsp90 paralogs are composed of an N-terminal ATP-ase domain, the client-binding middle domain, and a C-terminal dimerization domain [4]. The hallmark of the still incompletely understood chaperone cycle is the closure of the dimeric ATP-bound Hsp90 N-terminus over its substrates, which are released upon ATP hydrolysis [1,5]. Various co-chaperones of the cytoplasmic Hsp90 isoforms are known, which regulate ATPase activity and conformational transitions of the chaperone complex, and define client selectivity and recruitment [6].

The co-chaperone Cdc37 predominantly recruits kinases by thermodynamically challenging kinase conformational stabilities with its extreme N-terminus [2,7]. Only clients can subsequently associate with the C-terminus of Cdc37 in a 1:1 complex. This metastable complex is then efficiently recruited via the middle and C-terminal regions of Cdc37 to the N-terminal domain of Hsp90 for the conformational maturation of the open-state of the kinase [8]. Several conformational rearrangements of the complex create additional interfaces between the proteins [5,9]. The maturation lowers the affinity for Cdc37 and liberates the kinase and the chaperone machinery. In addition, dimeric Cdc37 arrests the chaperone cycle during client loading, before the ATP-dependent closure of the N-terminus of Hsp90, and therefore has a general effect on the overall Hsp90 activity [8,9].

In the 1990s, Hsp90 was identified as the target of the ansamycin geldanamycin and the resorcinol-moiety containing radicicol, two natural products with significant anti-cancer activity [10]. These findings stimulated interest in Hsp90 as a therapeutic target in cancer. The predominant rationale for the anti-cancer application is that Hsp90 inhibition leaves oncogenic driver kinases unfolded, upon which point they become proteasomally degraded. Most of the available Hsp90 inhibitors target the N-terminal ATP-binding pocket in all Hsp90 isoforms [11]. The ATP-binding mode is unusual, with the base and sugar buried in the pocket, and a kinked conformation of the ATP, which allows for selective inhibitor design [1].

The first generation geldanamycin derivatives, such as 17-allylamino-17-demethoxy-geldanamycin (17-AAG, tanespimycin), were readily tested in preclinical and clinical studies in several cancer types, but their development was ultimately discontinued [12]. Specifically, the liver toxicity linked to the reductive metabolism of the benzoquinone moiety appeared to be restrictive [12,13]. Resorcinol and purine-scaffold inhibitors, such as AUY922 (luminespib), displayed more promising pharmacological properties. Likewise, rationally-designed synthetic purine-scaffold Hsp90 inhibitors have generally greater potency and less toxicity [10,11]. However, dose-limiting toxicity remains an issue, and may be intrinsic to ATP-competitive inhibitors [14], because these induce the heat shock response, which upregulates proteins like Hsp70 and Hsp90 itself, thus fueling a vicious cycle that demands ever greater inhibitor concentrations [15].

Therefore, alternative Hsp90 drug-targeting strategies are needed. One possibility is to inhibit only one of the Hsp90 isoforms, such as Grp94 [16]. Another possibility is to target only co-chaperones [11], which implicitly focuses on the co-chaperone dependent cytoplasmic Hsp90 isoforms. While the targeting of the interactions of the Hsp90/Cdc37 complex with its client proteins is being explored [17], the inhibition of the Hsp90/Cdc37 interaction itself represents a relatively broader-acting approach.

This approach is exemplified by conglobatin A (also referred to as FW-04-806), a polyketide which binds to the N-terminus of Hsp90, which appears to disrupt the critical interaction of Arg167 on the C-terminus of Cdc37 with Glu47 of Hsp90 [18,19]. This compound inhibits the growth of HER2+ breast cancer cell lines with micromolar activity in vitro, and as a single agent in mouse SK-BR-3 and MCF-7 breast cancer-cell–derived xenografts [19,20]. As with ATP-competitive inhibitors, conglobatin A treatment leads to a degradation of Hsp90 clients, with G2/M cell cycle arrest and the induction of apoptosis [19]. Given that conglobatin A is particularly well tolerated in acute toxicity tests even at 900 mg/kg in mice [19], the targeting of the Hsp90/Cdc37 interface may represent a safer route for Hsp90 inhibition than the targeting of the ATP pocket.

Several other natural products were shown to either bind to the middle domain (kongensin A) or to the C-terminus of Hsp90 ((−)-epigallocatechin-3-gallate (EGCG), celastrol, and withaferin A), thus allosterically inhibiting the Hsp90/Cdc37 interaction [21,22,23,24,25]. However, their exact binding mode is often not fully characterized. Recently, the first small molecule inhibitors targeting the Hsp90/Cdc37 interface (K_d_ = 2.17 μM) were developed [26]. The binding mode of the tool compound DDO-5936 to Hsp90 (K_d_ = 3.86 μM) was resolved using nuclear magnetic resonance (NMR) spectroscopy and mutagenesis, revealing a major engagement of Glu47, Arg46 and Gln133 of Hsp90, which directly interferes with residues previously identified to be critical for the Hsp90/Cdc37 interaction [18]. Further improvement for affinity and pharmacological properties led to an orally-available compound referred to as 18 h, with a submicromolar affinity to Hsp90 (K_d_ = 0.5 μM) [27]. Despite their apparent proximity to the ATP-binding pocket, no disruption of ATP-pocket binders was observed. In line with this, there was no induction of the heat shock response. Importantly, both inhibitors were well tolerated, and inhibited the proliferation of HCT116 colorectal cancer cells in vitro (IC_50_(DDO-5936) = 9 μM; IC_50_(18 h) = 1.7 μM) and of derived xenografts in mice (40–50 mg/kg/d).

The successful translation of any drug candidate into the clinical setting critically depends on predictive biomarkers that explain the compound’s efficacy, and which can later help to select the sensitive patient population [28]. The overexpression of both Hsp90 and its co-chaperone, Cdc37, has been observed in breast, pancreatic, prostate, colorectal and hepatocellular cancers [4]. Indeed, the overexpression of Cdc37 under the control of the MMTV (mouse mammary tumor virus) promotor acts as a weak oncogene in mice [29]. Additionally, several cancer drivers that are Hsp90/Cdc37 client proteins—for example, hormone receptors, HER2, BCR-ABL, B-Raf and Src—were to some extent demonstrated to predict the response to drug treatment [10]. Encouraging clinical responses could also be seen for 17-AAG in HER2+ breast cancer patients, in combination with conventional trastuzumab therapy [30]. Interestingly, triple-negative breast cancers also respond to Hsp90 inhibitors [31]. Clinical responses are further observed in ALK-rearranged non-small cell lung cancer, but not consistently in many of the other cancer types that were expected to respond [10]. Altogether, these data suggest an incomplete rationale for the way in which to test and apply Hsp90 inhibitors.

So far, Ras signaling has not been mechanistically linked to Hsp90 activity. We recently identified conglobatin A as a selective disruptor of K-Ras4B- (hereafter K-Ras), but not H-Ras–nanocluster signaling complexes [32]. Recent evidence suggests that nanoclusters are composed of multiple units of stacked dimers of Ras and Raf [33,34,35,36]. Accordingly, nanoclustering strictly correlates with MAPK-signal output [37]. Nanoclusters can be further stabilized in a Ras isoform-specific manner, such as by galectin-3 (K-Ras) or galectin-1 (H-Ras), probably by the ability of these proteins to stabilize specific dimers of Raf proteins [33,38].

Here, we describe the molecular mechanism of the ways in which conglobatin A selectively inhibits K-Ras, which provides us with a novel biomarker rationale for the application of Hsp90 inhibitors. We then go on to identify and characterize several novel small molecule Hsp90/Cdc37 interface inhibitors with micromolar potencies. Our work supports a novel Hsp90 drug development and treatment rationale in multiple KRAS-driven cancer types.

## 2. Results

### 2.1. Conglobatin A Selectively Interferes with K-Ras Nanoclustering and Signaling by Inhibiting the Hsp90/Cdc37 Complex

**Figure 1:**
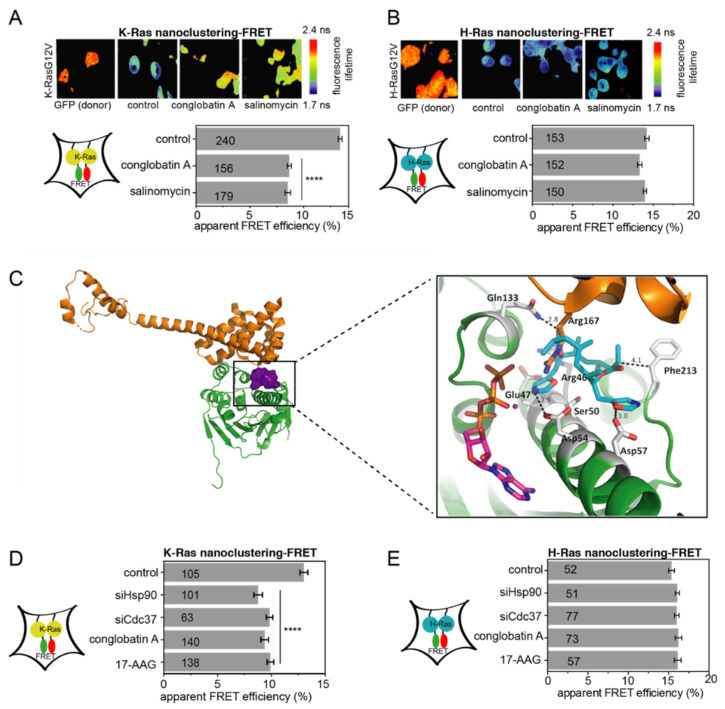
Conglobatin A selectively targets K-Ras via the inhibition of Hsp90/Cdc37. (**A**,**B**) FLIM-FRET images of HEK cells transfected with FRET-pairs of mGFP-/mCherry-K-RasG12V (**A**, top) or mGFP-/mCherry-H-RasG12V (**B**, top). The labelling indicates donor-only control or treatments. A decrease in the fluorescence lifetime indicates an increase in FRET due to nanoclustering. The transfected cells were treated with 0.1% DMSO vehicle control, 2 μM conglobatin A, or 1.3 μM salinomycin for 24 h. The nanoclustering-FRET analysis by treatment for K-RasG12V (**A**, bottom) or H-RasG12V (**B**, bottom). The numbers in the bars indicate the number of analyzed cells; *n* = 3 independent biological repeats. (**C**) Conglobatin A (purple surface) was docked into the crystal structure of human N-Hsp90 (green; PDB ID 3T0Z). The docked complex was overlaid on top of the crystal structure of yeast N-Hsp90 in a complex with human Cdc37 (PDB ID 1US7), of which only Cdc37 is depicted (orange). The magnification shows the details of how conglobatin A (cyan sticks) sterically blocks and disturbs critical interactions, notably between Glu47 of Hsp90 (gray sticks) and Arg167 of Cdc37 (orange sticks) at the interface of the complex. ATP is shown as magenta sticks, and magnesium is shown as a deep purple sphere. The polar interactions between conglobatin A and Hsp90 are shown as dashed lines, with distances in Ångströms. (**D**,**E**) The K-RasG12V- (**D**) and H-RasG12V- (**E**) nanoclustering-FRET in HEK cells co-transfected with mGFP- and mCherry-tagged RasG12V, together with 50 nM siRNA Hsp90, siRNA Cdc37, or 50 nM scrambled siRNA for compound/vehicle (control) treated samples. The next day, the cells were treated for 24 h with 0.1% DMSO vehicle control, 2 μM conglobatin A, or 2 μM 17-AAG. The numbers in the bars indicate the number of analyzed cells. The number of independent biological repeats *n* = 3 for K-Ras and *n* = 1 for H-Ras. **** *p* < 0.0001.

Ras nanoclusters are Ras-isoform specific di-/oligomeric nanoscale proteo-lipid signaling complexes [39]. Due to the dense packing in nanoclusters, Ras proteins tagged with Förster resonance energy transfer (FRET)-fluorophores exhibit a high FRET-level when they are expressed in cells. Nanoclustering can be isoform-selectively modulated, such as by the small molecule salinomycin, which blocks K-RasG12V- (Figure 1A) [32], but not H-RasG12V-nanoclustering in HEK cells (Figure 1B). We previously found that conglobatin A eradicates cancer cells with stem-like characteristics, and rationalized this activity with the selective inhibition of K-RasG12V-, but not H-RasG12V-nanoclustering (Figure 1A,B) [32]. However, the exact mechanism of the way in which K-Ras-nanoclustering is selectively affected by conglobatin A is unknown.

Based on our new data, and in agreement with previous reports, conglobatin A disrupted the interaction between the N-terminus of Hsp90 and its co-chaperone Cdc37 in a mammalian cell lysate-based split *Renilla* luciferase assay at low micromolar concentrations (Table 1; Figure S1A–C) [19,20]. The main interactions that hold the Hsp90/Cdc37 complex together are between Glu47 and Asp54 on Hsp90, which bind to the Arg167 of Cdc37 and Gln133 on Hsp90 binding to Asp170 of Cdc37 [8] (Figure S1D). The computational docking of conglobatin A to the structure of human cytoplasmic Hsp90 alpha (ATP-bound N-terminal fragment, *HSP90AA1*) suggested H-bonding between conglobatin A and Asp54, and Asp57 and Gln133 (Figure 1C; Figure S1E). Thus, conglobatin A stretches across the essential interface with Cdc37, and the compound also partly engages Hsp90 residues that would otherwise mediate binding to Cdc37.

As is consistent with this mechanism, both the knockdown of cytoplasmic Hsp90α/β (*HSP90AA1/HSP90AB1*) and of Cdc37 selectively decreased K-RasG12V-, but not H-RasG12V-nanoclustering-FRET (Figure 1D,E). Given that the same selectivity was observed with ATP-competitive Hsp90 inhibitor 17-AAG, this suggests that the loss of one or more Hsp90 clients was responsible for this effect.

**Figure 2:**
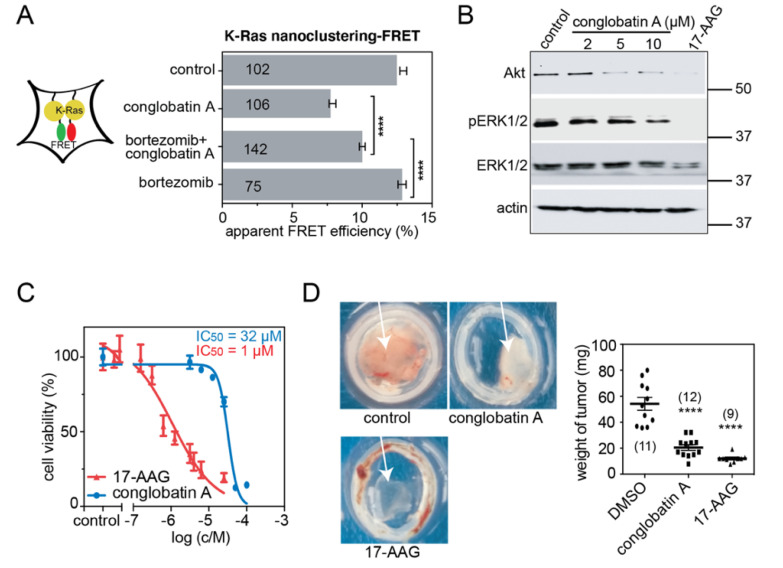
Conglobatin A inhibits Ras signaling, and cell- and microtumor-growth. (**A**) K-RasG12V nanoclustering-FRET in HEK cells co-transfected with mGFP- and mCherry-tagged K-RasG12V. The cells were pretreated with the DMSO vehicle control or 1 μM bortezomib for 2 h. Then, media containing conglobatin A or the vehicle control were added to the cells, to a final concentration of 2 μM or 0.1% DMSO, respectively. The cells were fixed after 24 h of this treatment. The numbers in the bars indicate the number of analyzed cells; *n* = 3 independent biological repeats. (**B**) Western blots of MDA-MB-231 cells that were treated with either 0.1% DMSO vehicle control or with the indicated concentrations of conglobatin A. In total, 2 μM 17-AAG served as the positive control. All of the drug treatments were for 24 h; *n* = 2 independent biological repeats. (**C**) 2D cell proliferation dose response curve of conglobatin A or 17-AAG, tested on MDA-MB-231 cells for 72 h; *n* = 2 independent biological repeats. (**D**) Microtumor formation of MDA-MB-231 cells on chicken CAM. The cells were treated with 0.1% DMSO vehicle control, 10 μM conglobatin A or 5 μM 17-AAG for 5 days; *n* = 3 independent biological repeats. **** *p* < 0.0001.

In agreement with the proteasome dependence of the Hsp90 client protein abundance, the co-treatment of HEK cells with the proteasome inhibitor bortezomib and conglobatin A incompletely, yet significantly, rescued the conglobatin A-induced reduction of K-RasG12V-nanoclustering-FRET (Figure 2A). In line with the strict correlation of K-Ras-nanoclustering with MAPK-signaling output [37], and with the effect of Hsp90 inhibitors on the abundance of Ras-effector kinases Raf and Akt [11], we found decreased pERK and Akt levels in KRAS-G13D mutant MDA-MB-231 triple-negative breast cancer cells upon treatment with conglobatin A (Figure 2B). Furthermore, the 2D proliferation of MDA-MB-231 cells was inhibited at IC_50_ = 32 ± 2 μM, while the clinically tested compound 17-AAG was much more potent (Figure 2C). Finally, we grew MDA-MB-231 into microtumors on the chick embryo chorioallantoic membrane (CAM). This cost- and time-efficient tumor model allows for the faithful analysis of many tumor features that are otherwise only accessible in mouse models [40]. Conglobatin A significantly reduced the microtumor growth in the model, with slightly lower efficacy than 17-AAG (Figure 2D).

These data collectively suggest that the inhibition of the Hsp90/Cdc37 interaction by conglobatin A decreases the abundance of one or more Hsp90 clients, which selectively affect K-Ras-nanoclustering and, concomitantly, MAPK signaling and tumor cell growth.

### 2.2. Hsp90 Inhibition Depletes the K-Ras Nanocluster Scaffold Galectin-3 Downstream of the Hsp90 Client HIF-1α

**Figure 3:**
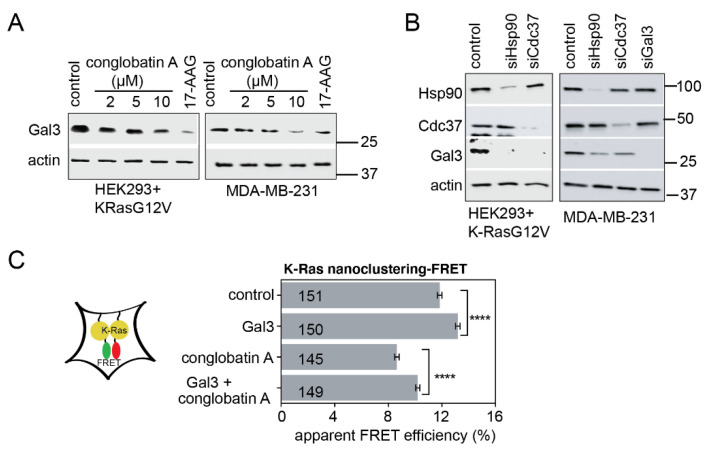
Conglobatin A depletes the K-Ras nanocluster scaffold galectin-3 via HIF-1. (**A**) Western blots of HEK cells transfected with mGFP-K-RasG12V, and of MDA-MB-231 cells treated with either 0.1% DMSO vehicle control or with the indicated concentrations of conglobatin A. In total, 2 μM 17-AAG served as the positive control. All of the drug treatments were for 24 h; *n* = 3 independent biological repeats. (**B**) Western blots of the HEK cells transfected with mGFP-K-RasG12V, and of MDA-MB-231 cells 48 h after knockdown using 50 nM for scramble control, siHsp90, siCdc37 or 25 nM for siGal3; *n* = 2 independent biological repeats. (**C**) K-RasG12V nanoclustering-FRET in HEK cells co-transfected with mGFP- and mCherry-tagged K-RasG12V. At the same time, Galectin-3 was expressed from 600 ng/mL pcDNA3.1-galectin-3 for 24 h. The cells were then treated with 2 μM conglobatin A for 24 h; *n* = 3 independent biological repeats. **** *p* < 0.0001.

It is well-established that expression of the nanocluster-scaffold galectin-3 (Gal3) selectively increases K-RasG12V-, but not H-RasG12V-nanoclustering [38]. We therefore tested whether the abundance of Gal3 could explain the K-Ras selectivity of conglobatin A. Indeed, conglobatin A dose-dependently reduced the Gal3 levels in K-RasG12V-transfected HEK and MDA-MB-231 cells (Figure 3A). Consistently, the knockdown of Hsp90 or Cdc37 in these cells significantly depleted the Gal3 levels (Figure 3B). Because Gal3 is not known as a client of Hsp90, an upstream transcriptional regulator of Gal3 and a client of Hsp90 were likely to be affected by Hsp90 inhibition. In agreement with such an indirect, transcriptional effect of Hsp90 inhibition on Gal3 levels, the overexpression of Gal3 partially rescued the conglobatin A mediated loss of K-RasG12V nanoclustering (Figure 3C).

**Figure 4:**
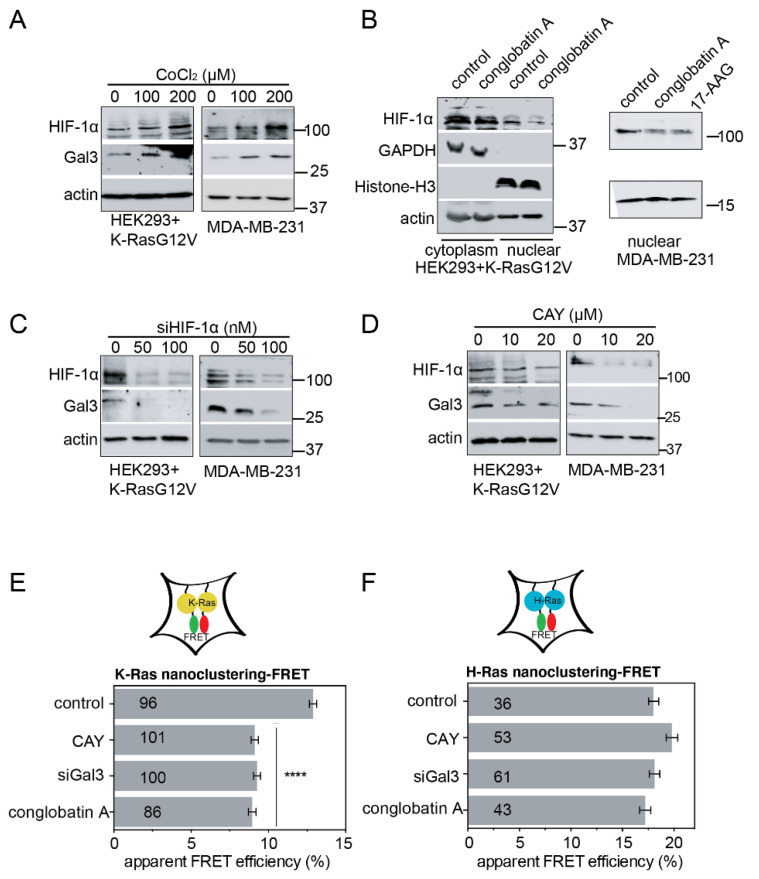
Conglobatin A depletes Hsp90 client HIF-1α and its transcriptional target galectin-3. (**A**) Western blots of HEK cells transfected with mGFP-K-RasG12V, and of MDA-MB-231 cells treated for 16 h with CoCl_2_; *n* = 1 independent biological repeat. (**B**) Western blots of cytoplasmic and nuclear extracts from HEK cells transfected with mGFP-K-RasG12V, and of MDA-MB-231 cells treated with either 0.1% DMSO vehicle control, 5 μM, or 10 μM conglobatin A, respectively. In total, 2 μM 17-AAG served as the positive control. All of the drug treatments were for 24 h; *n* = 1 independent biological repeat. (**C**) Western blots of HEK cells transfected with mGFP-K-RasG12V, and of MDA-MB-231 cells 48 h after the siRNA-mediated knockdown of HIF-1α; *n* = 2 for HEK and *n* = 1 for MDA-MB-231 independent biological repeats. (**D**) Western blots of HEK transfected with mGFP-K-RasG12V, and of MDA-MB-231 cells treated with 0.1% DMSO vehicle control or HIF-1α inhibitor CAY10585 (CAY) for 24 h; *n* = 1 independent biological repeat. (**E**,**F**) K-RasG12V- (**E**) and HRasG12V- (**F**) nanoclustering-FRET in HEK cells co-transfected with mGFP- and mCherry-tagged RasG12V, together with 50 nM siRNA-Gal3 or 50 nM scrambled siRNA for the compound/vehicle (control) treated samples. The next day, the cells were treated for 24 h with 0.1% DMSO, 2 μM conglobatin A, or 10 μM CAY10585 (CAY). The numbers in the bars indicate the number of analyzed cells. The number of independent biological repeats *n* = 2 for K-Ras and *n* = 1 for H-Ras. **** *p* < 0.0001.

Gal3 is induced by hypoxia, a condition suggested to support the emergence of cancer stem cell properties [41]. Specifically, HIF-1α, the master transcriptional regulator of the hypoxic response and a client of Hsp90 [42,43], is required for the induction and direct regulation of Gal3 expression [41,44]. Treatment with CoCl_2_, which mimics hypoxia [45], increased the HIF-1α levels and, concomitantly, Gal3 expression in K-RasG12V-transfected HEK and MDA-MB-231 cells (Figure 4A). Note that multiple HIF-1α-positive protein bands can be detected due to post-translational modifications, such as ubiquitination [46]. Conversely, the conglobatin A treatment, similarly to the 17-AAG treatment, decreased the nuclear levels of HIF-1α in these cells (Figure 4B). Moreover, the knockdown of HIF-1α (Figure 4C) or inhibition of HIF-1α transcriptional activity with CAY10585 (Figure 4D) decreased the Gal3 levels. In agreement with this, the inhibition of HIF-1α by CAY10585 selectively decreased K-RasG12V-, but not H-RasG12V nanoclustering-FRET to the same extent as the Gal3 knockdown or conglobatin A treatment (Figure 4E,F).

We therefore conclude that the inhibition of the Hsp90/Cdc37 interaction by conglobatin A reduces the expression of the K-Ras specific nanocluster scaffold Gal3, due to the depletion of its transcriptional regulator and Hsp90 client protein HIF-1α.

### 2.3. Computational and In Vitro Screening for Novel Hsp90/Cdc37 Interface Inhibitors

**Figure 5:**
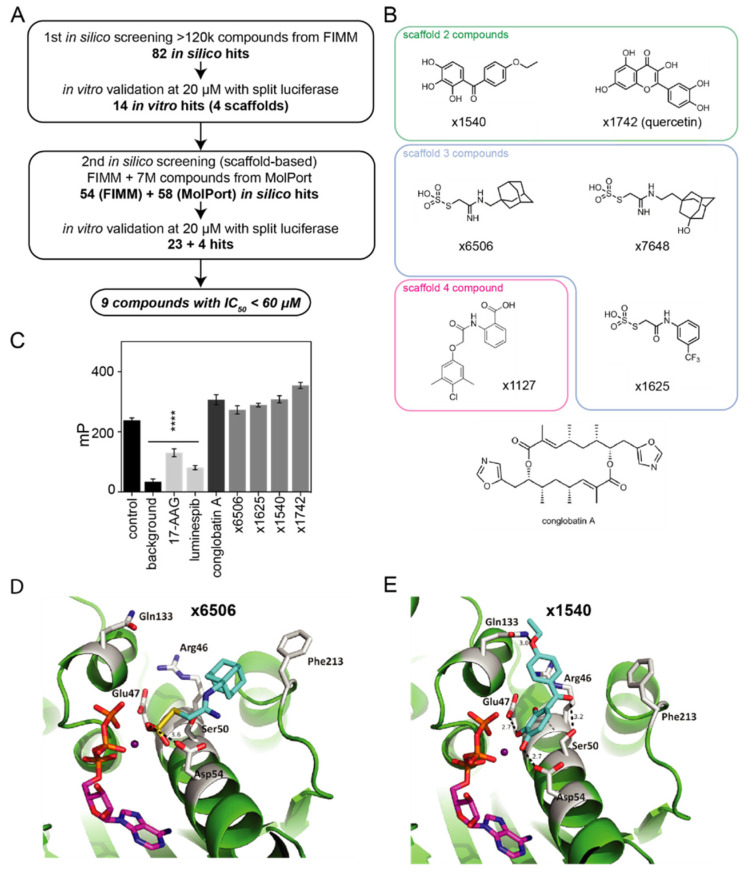
Screening for small molecule Hsp90/Cdc37 interface inhibitors. (**A**) Combined computational and in vitro compound screening workflow to identify novel small molecules with activities below 60 µM in the Hsp90/Cdc37 split *Renilla* luciferase assay. (**B**) Chemical structures of the selected top hit compounds, arranged by scaffolds. The structure of conglobatin A is shown for comparison at the bottom. (**C**) Competition with Hsp90 N-terminal ATP-binder FITC-geldanamycin decreases the polarization signal, indicating ATP-pocket binding (with 17-AAG, luminespib as positive controls). All of the compounds were tested at 20 µM. (**D**,**E**) The docked models for ×6506 (**D**) and ×1540 (**E**) in a complex with human N-Hsp90 (green; PDB ID 3T0Z) at the Hsp90/Cdc37 interface. Both compounds are shown as cyan sticks, the interacting residues in Hsp90 are shown as gray sticks, ATP is shown as magenta sticks, and magnesium is shown as a deep purple sphere. The polar interactions between the compounds and the protein are shown as dashed lines with distances in Ångströms. **** *p* < 0.0001.

Given the exceptionally low toxicity of conglobatin A compared to ATP-competitive Hsp90 inhibitors [19], the inhibition of the Hsp90/Cdc37 protein–protein interaction may represent a promising alternative strategy for future Hsp90 inhibitor development. We therefore computationally screened a library of >120,000 compounds from the Institute for Molecular Medicine Finland (FIMM), using a conglobatin A-based pharmacophore model and molecular docking (Figure 5A). Subsequently, the in silico hits were tested in vitro using the mammalian cell lysate-based split *Renilla* luciferase assay, which can distinguish Hsp90/Cdc37 interaction inhibitors from ATP-pocket competitors [25] (Figures S1C and S2A). This first in vitro validation led to the identification of four scaffolds (Figure S2B), which were used for a second round of in silico screening of the FIMM and approximately 7 M compounds comprising the MolPort compound libraries (a total of 112 in silico hits, 41 of which are available for repurchasing and in vitro testing) (Figure 5A; Data S1). After the in vitro validation, we identified nine compounds from three chemical scaffolds that significantly inhibited the interaction of the full length Hsp90 with Cdc37 at IC_50_ < 60 μM in the split *Renilla* luciferase assay (Data S1). We then immediately excluded large polyphenols, given their potential toxicity. The remaining six compounds were then confirmed to also inhibit the interaction of the N-terminal Hsp90 fragment with Cdc37, suggesting that they are true inhibitors of the intended interface (Figure 5B; Table 1; Figure S2C–F). Furthermore, all of the compounds had drug-like characteristics based on Lipinski’s rule of five, and showed no structural resemblance to conglobatin A. Notably, the most active compound ×6506 had IC_50_ = 4.1 ± 0.4 μM, an almost fivefold greater potency than conglobatin A.

We next tested two representative compounds from scaffolds 2 (×1540 and ×1742/quercetin) and 3 (×6506 and ×1625) (Figure 5B) for ATP-pocket binding. None of these four compounds displaced the ATP-competitive inhibitor geldanamycin at 20 μM (Figure 5C; Figure S3A), and the most active compounds from the two scaffolds, ×6506 and ×1540, did not do so up to 50 μM (Figure S3B,C). The comparison of the docking models of ×6506 (Figure 5D) and ×1540 (Figure 5E) suggested two distinct binding modes. While both compounds coordinated the Mg^2+^ ion that is also part of the ATP-pocket and Asp54, compound ×1540 made additional H-bonding contacts with Glu47, which is critical for binding to Arg167 from Cdc37. Moreover, Ser50 and Gln133 (H-bond), as well as a π-cation interaction with Arg46, was suggested by the docking (Figure 5E). By contrast, ×6506 orients with its bulky adamantyl substituent toward a small hydrophobic pocket demarcated by Phe213 (Figure 5D). These two distinct binding modes were supported by the docking of the second best compounds from the same scaffold, ×1742 and ×1625, respectively, albeit ×1625 seems to adopt a mixed pose, with its trifluormethyl-benzyl group oriented only partly toward Phe213 (Figure S3D,E). The comparison of the potencies of ×6506 and ×1625 (Table 1) suggested that the large adamantyl substituent near Phe213 was advantageous, possibly also due to having a stronger sterical impact on the Hsp90/Cdc37 interface.

In summary, we identified four new small molecules that disrupt the Hsp90/Cdc37 interface with potencies between 4 μM–44 μM, while not binding to the ATP-pocket. Our docking models suggest that three different sub-sites on the surface are engaged.

### 2.4. Both ×6506 and ×1540 Deplete HIF-1α and Gal3 to Selectively Affect K-Ras

Hsp90 inhibitors have been tested in a number of different cancer types, in particular in kinase-dependent breast cancer cell lines, such as Her2-overexpressing SK-BR-3 cells. However, based on our molecular mechanism that explains K-Ras targeting, we included in our cellular analysis KRAS mutant, HIF-1α high, and/or Gal3 high cancer types, such as pancreatic and lung adenocarcinoma (Figure S4A,B). Importantly, high HIF-1α levels and, to some extent, high Gal3 expression are associated with a poorer survival of human cancer patients, in particular in association with mutant KRAS, which emphasizes the need for suitable potent inhibitors (Figure S4C–F). Using the ATARiS dependency data from the RNAi screen in Project DRIVE [47], we selected—in addition to SK-BR-3—three KRAS-mutant cell lines (MDA-MB-231, MIA PaCa-2 and HCC44) with various degrees of dependency on KRAS, Gal3 and HIF-1α (Figure S4G) for the further evaluation of our compounds.

**Figure 6:**
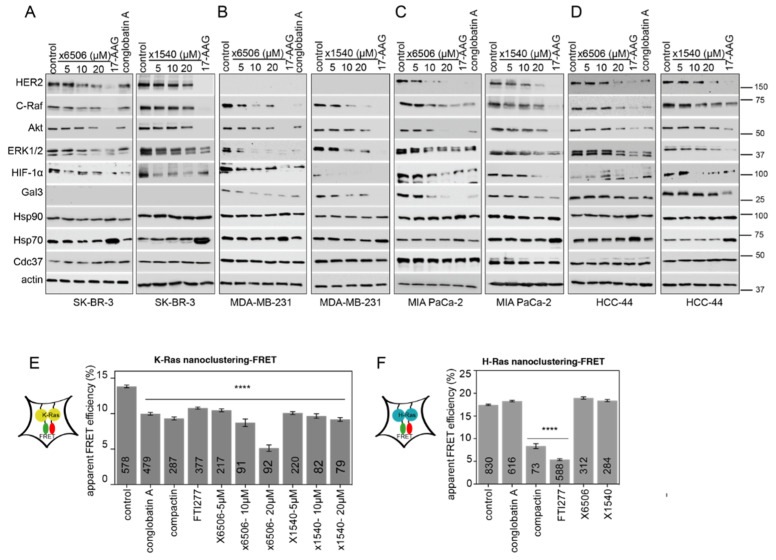
Top compounds decrease the expression of Hsp90 clients, and dose-dependently inhibit K-Ras nanoclustering-FRET. (**A**–**D**) Western blots of HER-2 overexpressing SK-BR-3 (**A**), KRAS mutant triple negative breast MDA-MB-231 (**B**), KRAS mutant pancreatic MIA PaCa-2 (**C**), and KRAS mutant lung HCC-44 (**D**) cancer cell lines. The cells were treated with 0.1% DMSO vehicle control or the indicated concentration of compounds. In total, 2 μM 17-AAG or 10 μM conglobatin A served as the positive control. Note the increase in Hsp90 and, in particular, Hsp70 with the 17-AAG treatment, which was not observed with interface inhibitors. All of the drug treatments were for 24 h; *n* = 1 independent biological repeat. (**E**,**F**) K-RasG12V- (**E**) and HRasG12V- (**F**) nanoclustering-FRET in HEK cells co-transfected with mGFP- and mCherry-tagged RasG12V. The cells were treated for 24 h with 0.1% DMSO vehicle control, 2 μM conglobatin A, 2 μM compactin, 0.5 μM FTI277, or the indicated concentrations of ×6505 or ×1540 (**E**) or 5 μM of these hit compounds (**F**). Compactin and FTI277 block Ras prenylation and farnesylation, respectively, and served as positive controls. The numbers in the bars indicate the number of analyzed cells; *n* = 3 independent biological repeats. **** *p* < 0.0001.

The inhibitor activity in cells is visible by the depletion of Hsp90 clients, such as kinases. We therefore examined the effect of ×6506 and ×1540 on kinases (Her2, C-Raf, Akt, ERK1/2), on additional proteins relevant in our context (HIF-1α, Gal3), and on Cdc37, Hsp90 and Hsp70 levels. The expression of the latter two is increased in the HSF1-mediated activation of the so-called ‘heat shock response’ by Hsp90 ATP-pocket inhibitors, such as 17-AAG [12]. Eventually, this induction requires ever higher levels of inhibitor, limiting its efficacy. Importantly, this response is absent with some of the Hsp90/Cdc37 interaction inhibitors [22,26]. In agreement with their activity as Hsp90/Cdc37 interface inhibitors, both of our novel inhibitors dose-dependently decreased the expression of established Hsp90 clients (C-Raf, Akt, ERK), while not inducing the expression of Hsp90 or Hsp70 in the four cancer cell lines, unlike 17-AAG (Figure 6A–D). In general, the HIF-1α levels decreased with the compound treatment, and, concomitantly, the Gal3 expression was lost (Figure 6B,C). This loss of Gal3 appeared less pronounced in HCC-44 cells, which could be due to other additional regulators than HIF-1α, such as NF-κB, maintaining their expression in that cell line [48] (Figure 6D). Using the nanoclustering-FRET assays, we further confirmed that x6506 and x1540 displayed K-Ras-selectivity like conglobatin A in HEK cells (Figure 6E,F). The same was also true for the other two compounds from the same scaffold, supporting our hit identification strategy (Figure S4H,I).

Collectively, these data demonstrate the micromolar on-target cellular activity of the selected compounds ×6506 and ×1540.

### 2.5. Compounds ×6506 and ×1540 Decrease Cancer Cell Proliferation, and Spheroid- and Microtumor-Formation

**Figure 7:**
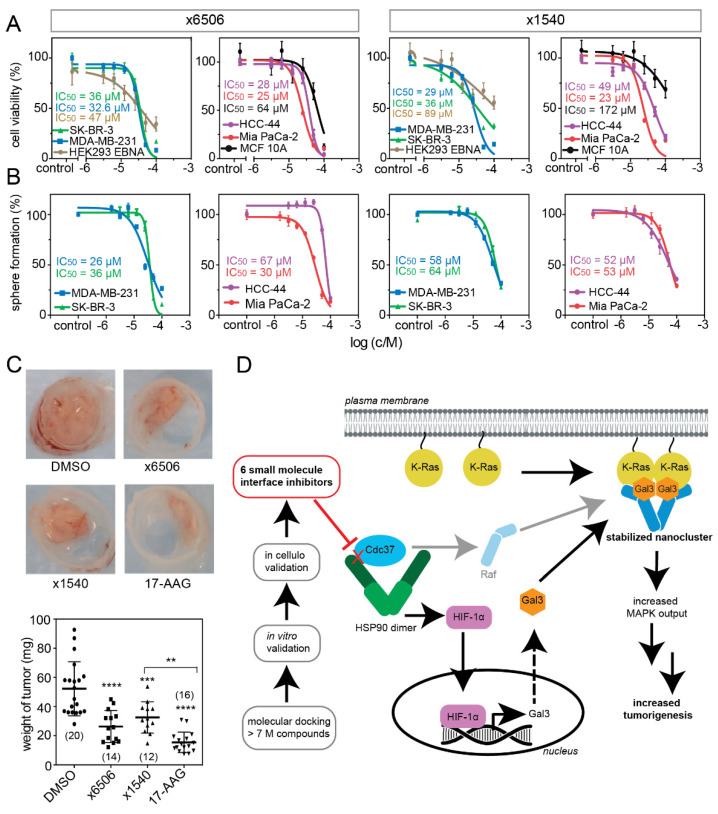
Top compounds inhibit 2D and 3D cancer cell growth and microtumor formation in the CAM model. (**A**) Compounds ×6506 or ×1540 dose-dependently reduced the 2D proliferation of several cancer and non-transformed cell lines after 72 h; *n* = 3 independent biological repeats. (**B**) Compounds ×6506 or ×1540 dose-dependently reduced the 3D spheroid growth of several cancer cell lines cultured under low attachment and serum-free conditions after 72 h; *n* = 3 independent biological repeats. (**C**) Microtumor formation of MDA-MB-231 cells on chick embryo CAM. The cells were treated with 0.1% DMSO vehicle control, 20 μM ×6506 or ×1540, or 10 μM 17-AAG for 5 days; *n* = 2 independent biological repeats. The number of analyzed microtumors per condition is given in brackets in the plot. (**D**) Summary scheme showing, on the left, the compound screening success, leading to six new small molecule Hsp90/Cdc37 protein interface inhibitors that could serve as a starting point for drug development. The major part on the right illustrates how Hsp90 activity is linked to the K-Ras signaling output via HIF-1α and Gal3, which can stabilize the K-Ras nanocluster and thus increase the MAPK-output. According to our nanocluster model, Raf proteins are integral components; hence, we here indicate a potential mechanistic contribution via Raf. However, it is currently unknown which Raf-dimers preferentially stabilize the K-Ras nanocluster. ** *p* < 0.005, *** *p* < 0.001, **** *p* < 0.0001.

We next analyzed the inhibition of the 2D growth of the breast, pancreatic, and lung cancer cell lines (Figure 7A). A somewhat-higher activity of ×6506 and ×1540 was found in the pancreatic MIA PaCa-2 cell line (Table 2). However, altogether, the IC_50_ values were quite similar, ranging between 23 μM and 49 μM, which was comparable to the potencies found in vitro (Table 1). When examining the activity of the compounds in these cells grown as 3D spheroids under low adherent, serum-free conditions (Figure 7B), we found activities between 26 μM and 67 μM, with ×6506 having a higher activity than ×1540, except for in HCC-44, as would be expected from their in vitro potency relation. The activity was also, overall, the lowest in HCC-44, which is consistent with the more limited effect of the compounds on Gal3 levels (Figure 6D).

The other two compounds, ×1625 and ×1742 (quercetin) did not decrease the spheroid formation in these cancer cell lines at 20 μM (Figure S5A–D). By contrast, quercetin (×1742) significantly increased the spheroid numbers in MDA-MB-231 and MIA PaCa-2, indicating that this particular compound or chemotype could be problematic, due to off-target effects (Figure S5B,C). Overall, the MDA-MB-231 3D spheroids appeared to most sensitively capture the Hsp90/Cdc37 inhibitor activities (Table 3; Figure S5B). We therefore tested the effect of ×6506 and ×1540 on the microtumor formation of MDA-MB-231 cells in the CAM model. In agreement with the previous cellular data, both compounds significantly reduced the microtumor growth to a similar extent to 17-AAG (Figure 7C).

## 3. Discussion

Our results provide a mechanistic explanation for the ways in which the inhibition of Hsp90, such as by blocking its interaction with its co-chaperone Cdc37 with conglobatin A, selectively disrupts K-RasG12V-, but not H-RasG12V nanoclustering and signaling. This selectivity profile is important, as it enables the selective targeting of stemness traits in cancer cells [32,49]. Mechanistically, Hsp90/Cdc37 interface inhibition not only affects kinase maturation, but broadly disrupts Hsp90 activities, including the stabilization of HIF-1α. This is because Cdc37 also more generally affects the Hsp90 chaperone cycle [8,9]. The loss of HIF-1α in the nucleus decreases the expression of its target Gal3. As Gal3 specifically stabilizes the GTP-K-Ras nanocluster, its loss only abrogates K-Ras-nanoclustering and the ensuing MAPK signal output [38] (Figure 7D).

In agreement with our mechanistic data, it has been shown that the Hsp90/Cdc37 interaction inhibitor celastrol negatively regulates the HIF-1α pathway [50]. Similarly, an inhibitor of casein kinase 2 (CK2), which modulates the activity of Hsp90/Cdc37, was found to downregulate HIF-1α [51]. Moreover, the importance of HIF-1α in Ras/MAPK signaling was previously noted for Her2 positive cancers, which require HIF-1α for anchorage independent growth [52]. Therefore, Hsp90 inhibition will be particularly relevant in HIF-1α and/or Gal3-high tumors, a biomarker context that is most frequently found in pancreatic and lung cancers.

The future efficacy testing of Hsp90 inhibitors may therefore benefit from an assessment in these cancer types and in selected tumors with the corresponding biomarker signatures. Indeed, the Hsp90/Cdc37 inhibitors celastrol, (−)-epigallocatechin-3-gallate (EGCG, identified also in this study), and withaferin A were previously successfully tested against pancreatic cancer cell growth in vitro and in vivo by the Sun group [22,24,53]. However, in their case, pancreatic cancer cells were selected due to the ‘complex nature’ of pancreatic cancer [24]. In line with the Hsp90 connection to K-Ras, a synergistic activity of Hsp90 inhibitors with MAPK-pathway inhibitors in KRAS mutant pancreatic ductal adenocarcinoma was reported [54]. Moreover, recent proteomics data suggest that the protein–protein interaction networks of K-Ras mutant colorectal cancer cells are rewired to enhance the connectivity to Hsp70 and Hsp90 family proteins [55].

Our molecular mechanism-based K-Ras focus contrasts with previous strategies to select cancer types for treatment with Hsp90 inhibitors. For instance, the expression of Hsp90 or Cdc37 was used to identify potentially-responsive tumor types. While the Hsp90 isoform levels vary in tumors, and sometimes correlate with drug resistance or tumor stage [4,56], high expression levels of Cdc37 are more consistently found in proliferating cancer cells [57]. Given that Hsp90 inhibition affects multiple clients that are established cancer drug targets, it is obvious that we do not exclude the relevance of other clients for the observed anti-cancer activity. In particular, Raf proteins may be mechanistically connected, as our nanocluster model suggests that Raf proteins scaffold nanocluster, and that this complex can be further stabilized by galectins [33,58] (Figure 7D). However, the current models of Ras–Raf interaction cannot explain why the selective depletion of one or two specific Raf paralogs could selectively affect only one Ras isoform, as we observe. Interestingly, many combination therapy approaches these days aim to hit several targets in one pathway, such as Raf and MEK [59]. Based on our new mechanism, the inhibition of Hsp90 emulates this by hitting K-Ras (in particular in HIF-1α–high, Gal3-high cancers) in addition to Raf and ERK. This may explain a pronounced drug action via the MAPK pathway.

Another important set of clients of Hsp90 are mutants of the tumor suppressor p53 [60]. Incidentally, all of the cancer cell lines investigated here also carry mutations in p53 [61]. Therefore, Hsp90 inhibition may also, via p53, affect cell survival. Clearly, a future study of a broader palette of cancer cell lines with more matured interface inhibitors would allow us to pinpoint several important signaling pathways downstream of Hsp90.

We further identified novel micromolar active Hsp90/Cdc37 interface inhibitors as promising starting points for further Hsp90 drug development. Hsp90/Cdc37 interface inhibition represents an alternative strategy to target Hsp90, but without the problematic induction of the ‘heat shock response’ which is seen with ATP-pocket inhibitors. Based on conglobatin A toxicity data and the recently-published small molecule inhibitor (18h) [27], the targeting of the Hsp90/Cdc37 interface may be pharmacologically advantageous. The here-identified drug-like small molecules from different scaffolds may thus help to further validate the Hsp90/Cdc37 interface for its low on-target toxicity. In this context, it is noteworthy that we concentrated on the interface with the N-terminus of Hsp90, while other approaches looked at the interface between Cdc37 and the middle domain of Hsp90 [62,63]. While the computational and experimental structural data from us and others identified novel small molecule inhibitors binding to a Hsp90 surface in the vicinity of the ATP-binding pocket, they did not disrupt ATP-pocket binding [26]. Based on the observation that the silencing of Cdc37 sensitizes cancer cells to ATP-pocket binding Hsp90 inhibitors, combinations of the Hsp90/Cdc37 interface and ATP-pocket inhibitors could act synergistically [64].

Our docking data of conglobatin A to the Hsp90/Cdc37 interface are in agreement with recent studies that highlighted the significance of the N-terminal hydrophobic surface around the H2 helix of Hsp90 [19,26]. While conglobatin A occupies critical residues that mediate the Hsp90/Cdc37 interaction, its extension across the whole interaction interface suggests a significant sterical component of its inhibitory activity. This sterical component seems to be emulated by the adamantly-containing compound ×6506 that we have discovered. Like conglobatin A, the more recent inhibitors DDO-5936 and compound (18 h), and our here-identified compounds engage residues Glu47 and Gln133 on Hsp90, which are otherwise critical to stabilize the complex with Cdc37 [26,27]. The currently most potent small molecule Hsp90/Cdc37 interface inhibitor, compound (18 h) (K_d_ = 0.5 μM) was more efficacious in reducing the growth of HCT116 cell-derived colorectal cancer xenografts than its predecessor, DDO-5936. Given that the latter has a similar low micromolar inhibitory activity on the complex to our best inhibitor ×6506, it is probable that our current hits need further improvements for successful in vivo application.

Currently, PROteolysis-Targeting Chimeras (PROTACs) are under heavy development for difficult targets, as they do not have to bind to an active site [65]. PROTACs potentially offer a more selective opportunity to deplete a certain protein, compared to the broad effects of Hsp90 inhibition. Nevertheless, PROTACs require the same pharmacological optimization as other inhibitors. Broader-acting inhibitors, such as Hsp90 inhibitors, typically prevent resistance development. In a sense, Hsp90/Cdc37 inhibitors can therefore be regarded as kinome-/Hsp90-ome-wide PROTACS. A shared beneficial property of both PROTACs and Hsp90 inhibitors is their potential to eliminate not only excess enzymatic activity, but also the scaffolding functions of kinases, such as of Raf [66].

Besides cancer, Hsp90 inhibitors have been tested in other diseases, including those caused by protozoans [67]. Another recently-evolving application is the use of Hsp90 inhibitors as a novel class of drugs that selectively eliminate the senescent cells (senolytics) which accumulate in aging mammals, and are thought to drive a systemic inflammatory condition that accelerates aging [68]. Therefore, senolytics are used in longevity therapy to extend the life- and health-span, and quercetin—in combination with the tyrosine kinase-inhibitor dasatinib—is currently under clinical investigation for a senolytic effect in several aging-associated diseases [69] (e.g., NCT04063124, NCT02874989). Intriguingly, we here identified quercetin (×1742) to be active at approximately 40–50 µM against Hsp90/Cdc37. This specific activity was not previously known, albeit that Hsp90 was identified as a target of quercetin in a photoaffinity labelling approach [70]. Therefore, our data are in line with the identification of Hsp90 inhibitors as a good candidate for senolytics [68]. It furthermore suggests that at least part of the senolytic activity of quercetin relates to Hsp90 inhibition. However, given the spheroid-promoting activity that we observed in some KRAS mutant cancer cell lines, this compound could be problematic if these observations translated into the patient situation.

In the light of the anti-cancer and anti-aging potential of Hsp90 inhibitors, Hsp90/Cdc37 interface inhibitors warrant further development, given their potentially low on-target toxicity.

## 4. Materials and Methods

### 4.1. DNA Constructs and siRNA

The plasmids pmGFP-/mCherry-H-RasG12V and pmGFP-/mCherry-K-RasG12V were previously described [71,72]. pcDNA3.1-galectin-3 [38] was used for the overexpression of human galectin-3. pcDNA3.1(+)-NRL-Hsp90 and pcDNA3.1(+) CRL-Cdc37 were previously described [18]. pcDNA3.1(+)-NRL-N-Hsp90 was constructed by PCR amplifying from the original pcDNA3.1(+) −NRL-Hsp90 plasmid by a forward primer designed with a BamHI site (5′GTT GTT GGATCC A ATG CCT GAG GAA ACC CAG ACC3′) and a reverse primer designed with a XhoI site (5′CCG CCG CTCGAG TTA CTA CTC CAC AAA AAG AGT AAT G3′). The amplified product, corresponding to residues 1–223 of Hsp90, was cloned into the same plasmid pcDNA3.1(+), after digestion with the corresponding restriction enzymes (BamHI and XhoI).

For the silencing of human Hsp90, Cdc37, galectin-3 and HIF-1α, we used siRNA Hsp90 (cat. no. sc 35608), siRNA Cdc37 (cat. no. sc 29255) and siRNA galectin-3 (cat. no. sc 155994) from Santa Cruz Biotechnology. The siRNA HIF-1α sequence 5′-AAC UAA CUG GAC ACA GUG UGU(dTdT)-3′, described in [73], was obtained from Eurofin Genomics. ON-TARGETplus, a non-targeting siRNA (cat. no. D 001810-10-05) from Dharmacon, was used as a control for the siRNA knockdown. The transfection reagent jetPRIME (cat. no. 114-75) was purchased from Polyplus. The jetPRIME was used for the transfections in the FLIM-FRET experiments, and the siRNA transfections in HEK and MDA-MB-231 cells. FuGENE HD (cat. no. E2311, Promega) was initially used for the transfections in the FLIM-FRET experiments. All of the transfections were performed according to the manufacturers’ protocols.

### 4.2. Chemical Compounds and Inhibitors

The conglobatin A (cat. BIAC1022) and salinomycin (cat. no. BIA-S1307) were purchased from BioAustralis. The CAY10685 (cat. no. sc-205346), 17-AAG (cat. sc-200641), and cobalt-(II) chloride (CoCl_2_) (cat. no. sc-252623) were purchased from Santa Cruz Biotechnology. The epigallocatechin-3-gallate (EGCG; cat. 70935) was purchased from Cayman Chemical. The geldanamycin (cat. T6343) was obtained from TargetMol, luminespib (cat. HY-10215) from MedChemTronica, novobiocin (cat. N825320) was obtained from Toronto Research Chemicals, and withaferin A (cat. NP-007425) was obtained from AnalytiCon Discovery. The compactin (cat. no. M2537) was obtained from Sigma Aldrich. The FTI277 (cat. no. 344555) was from Calbiochem. The bortezomib (cat. no. 2204S) was obtained from Cell Signaling Technology. The sources of the repurchased hit compounds are provided in Data S1.

### 4.3. Cell Culture

The HEK293 EBNA (HEK) cells were a kind gift from Prof. Florian M Wurm, Swiss Federal Institute of Technology Lausanne (EPFL), Lausanne, Switzerland. The MDA-MB-231 (HTB-26), SK-BR-3 (HTB-30) and MIA PaCa-2 (CRM-CRL-1420) were obtained from American Type Culture Collection (ATCC). The HCC-44 (ACC 534) were obtained from German Collection of Microorganism and Cell Culture (DSMZ). The HEK and MIA PaCa-2 were cultured in Dulbecco’s modified Eagle’s medium (DMEM, cat. no. D6171, Sigma Aldrich), supplemented with 10% fetal bovine serum (FBS) (cat. no. S1810, Biowest) and 2 mM L-glutamine (cat. no. G7513, Sigma-Aldrich). The MDA-MB-231, SK-BR-3 and HCC-44 were maintained in RPMI (cat. no. R586, Sigma-Aldrich), containing 10% FBS and 2 mM L-glutamine. All of the cell lines were subcultured twice a week and incubated at 37 °C with 5% CO_2_ in a humidified cell incubator.

### 4.4. Fluorescence Lifetime Imaging Microscopy (FLIM)-FRET

In total, 80,000 HEK cells were seeded in 12-well plates containing sterilized 16-mm coverslips and 1 mL medium. The next day, the cells were transfected using jetPRIME or FuGENE HD transfection reagent. For the donor fluorophore-only samples, the cells were transfected with 500 ng mGFP-tagged RasG12V plasmid. For the nanoclustering-FRET pair samples, the cells were transfected with both the donor and acceptor constructs, i.e., pmGFP- and pmCherry- RasG12V at a plasmid ratio of 1:3, respectively, and a total of 800 ng plasmids. The next day, the cells were treated with either 0.1% DMSO vehicle control or the indicated concentrations of compounds diluted in DMEM or RPMI medium for 24 h. For the gene silencing studies, the cells were transfected with the indicated amounts of siRNAs, together with the FRET pair constructs. The plasmids and siRNA were diluted in the same jetPRIME transfection buffer, followed by the addition of jetPRIME. After 48 h of transfection, the cells were fixed in 4% paraformaldehyde for 10 to 15 min, before being mounted onto slides with Mowiol 4–88 (cat. no. 81381, Sigma-Aldrich). The lifetime of the mGFP donor fluorophore was measured using a fluorescence microscope (Zeiss AXIO Observer D1) equipped with a fluorescence lifetime imaging attachment (Lambert Instruments), as described previously in detail [74]. The apparent FRET efficiency (E_app_) was calculated as E_app_ = (1 − τ_DA_/τ_D_) × 100%, with the measured fluorescence lifetimes τ_DA_ of the FRET sample and the τ_D_ of the donor-only sample.

### 4.5. Computational Compound Screening and In Vitro Hit Validation Workflow

The computational screening was performed using the compound database of 128,201 small molecules provided by the Institute for Molecular Medicine Finland (FIMM), and using the MolPort screening compound library, which has over 7 million commercially-available compounds (MolPort Inc.). The 3D structures of all of the compounds were constructed using Maestro software in the Schrödinger package (Maestro, 2018, Schrödinger Release 2018-4: Schrödinger, LLC, New York, NY, 2018). The geometry optimization of all of the compounds was performed using the OPLS3 force field and the Powell conjugated gradient algorithm method with convergence criterion of 0.01 kcal/(mol Å) and a maximum iterations set of 1000 [75].

The three-dimensional coordinates for the molecular structures of the examined proteins were retrieved from the Protein Data Bank (PDB; rcsb.org) [76]. All of the protein simulations were performed using the OPLS3 force field [75]. The protein structure was prepared for the simulations using the Protein Preparation Wizard in Schrödinger [77]. The 2.2 Å ATP-bound complex of the N-terminal domain of human Hsp90 (N-Hsp90) with an ‘open’ position of the ‘lid’ (PDB ID 3T0Z; [78]) was used for the in silico screening. The inhibitor binding site, in relation to the Cdc37 interaction surface, was determined by utilizing the crystal structure of yeast N-Hsp90 in a complex with the C-terminal domain of human Cdc37 (PDB ID 1US7; [8]).

The first round of virtual screening was performed with two parallel protocols using the programs within the Schrödinger package: (1) Conglobatin A was first docked to N-Hsp90 (PDB ID 3T0Z) with Glide using the extra precision (XP) mode [79]. This complex was then employed to construct a pharmacophore model, and the virtual screening was performed using the Phase program [80]; (2) the molecular docking-based virtual screening was performed with Glide [79]. Thereafter, the results were subjected to Glide docking simulations using the standard precision (SP) and the XP modes with ligand flexibility and the Epik state penalties for the docking score. The MM-GBSA free energy for binding was calculated for the XP-docked complexes using the VSGB 2.0 solvation model, allowing flexibility for the residues within 6.0 Å distance from the docked compound [81]. All of the visualizations were carried out using PyMOL (The PyMOL Molecular Graphics System, Version 2.0 Schrödinger, LLC).

In the first screening round of the FIMM compound library, we obtained 82 in silico hits, of which we confirmed 14 as in vitro hits using the full length Hsp90/Cdc37 split *Renilla* luciferase assay (at 20 μM; 10 of 14 repurchased). In the second in silico screening of the FIMM database, we employed the four identified chemical scaffolds (Figure S2B) for the visual inspection of the FIMM compound library. The identified 54 hits were docked using Glide XP docking [79] and evaluated. Of the 54 hits, 23 hits were validated in vitro at 20 μM. However, only four of them could be repurchased for further investigations. Therefore, we searched the MolPort online library (MolPort Inc., accessed October 2018) for compounds similar to the scaffolds using a SMILES-based search. Of the obtained 58 in silico hits, 37 could be purchased for in vitro testing. All of the purchased compounds were validated in vitro at 20 μM using the split *Renilla* luciferase assay.

### 4.6. Hsp90/Cdc37 Split Renilla Luciferase Interaction Assays for Compound Screening

The Hsp90/Cdc37 split *Renilla* luciferase assay using the full length Hsp90 for the compound screening was described previously [25]. In addition to this, we here established an analogous assay using only the N-terminal ATP-ase domain of Hsp90 (N-Hsp90, residues 1-223). In brief, individual batches of HEK cells were transfected with plasmid pcDNA3.1(+)-NRL-Hsp90, respectively, pcDNA3.1(+)-NRL-N-Hsp90, or pcDNA3.1(+)-Cdc37-CRL using jetPRIME. After 48 h, the cells were trypsinized and washed with PBS. The harvested cells were lysed in 1 mL of the 1x lysis buffer provided in the *Renilla* Luciferase Reporter Assay System Kit from Promega (cat. E2820), and were incubated on ice for 10 min. The cell lysates were then cleared by centrifugation at 13,000× *g* for 1 min. In total, 20 µL assay buffer from the afore mentioned kit was added to each well of a white 96-well flat bottom plate (cat. 3917, Costar, Corning Inc). After this, the screening/test compounds or DMSO—as indicated—were added to each well. In total, 5 µL NRL-Hsp90- or NRL-N-Hsp90-lysate was added to each well, one row at a time. Subsequently, the plate was incubated for 5 min at room temperature (RT). After the incubation, 5 µL Cdc37-CRL was added and incubated for 2 min at RT. Just before the reading, 20 µL assay buffer containing 2× *Renilla* luciferase substrate (coelenterazine) was added. For the FIMM screening, sample wells were read on a PHERAstar plate reader (BMG LABTECH) at a gain level of 4000, after 10 s shaking, using a hypomorph of NRL-Hsp90. For the subsequent validations of the compounds, sample wells with the above-described assay constructs were read using a synergy H1 Hybrid Multi-Mode reader (BioTek) in the luminescence detection mode, as described previously in detail [25].

### 4.7. Hsp90 N-Terminal ATP-Binding Site Competition Assay

In order to assess the activity of the compounds on the ATP-binding site of Hsp90, the competition with the labelled ATP-pocket inhibitor geldanamycin was measured by the use of the Hsp90α (N-terminal) Assay Kit (cat. no. 50298, BPS Bioscience), according to the manufacturer’s instructions. In brief, 5 nM FITC-labeled geldanamycin was mixed with 20 μM test compound in the presence of the kit’s assay buffer, 2 mM DTT (cat no. BP172, Thermo Fisher Scientific) and 100 μg/mL BSA (cat. no. A8022, Sigma-Aldrich). The reaction was initiated by adding 35 ng/μL purified recombinant Hsp90α, and was incubated at RT with slow shaking for 2 h. All of the reactions were performed in a black, low binding NUNC microtiter plate, included in the kit, and the total reaction volume was 50 μL. The enzyme control was measured without the test compound, and the enzyme background control was measured without the test compound or Hsp90α. The blank was measured without the FITC-geldanamycin, test compound or Hsp90α. All of the controls and the blank contained 10% DMSO, as did all of the samples of the test compounds. All of the samples were analyzed as duplicates. The fluorescence polarization of the samples was measured using a Synergy H1 Hybrid Multi-Mode reader with a polarization cube (excitation 485 ± 10 nm, emission 528 ± 10 nm). The blank value was subtracted from all of the other values, and the fluorescence polarization was determined as:

(1) $$\mathrm{mP}\;=\;(\left(I_{\mathrm{II}}\;-{\;I}_{⊥}\right)/\left(I_{\mathrm{II}}+{\;I}_{⊥}\right))\;\times \;1000$$

where I_II_ is the intensity of the parallel, and I_┴_ the intensity of the perpendicular polarized light.

### 4.8. Western Blotting

The cells were trypsinized, washed with PBS, and lysed in RIPA buffer (cat no. 89900, ThermoFisher Scientific) containing protease and phosphatase inhibitors (cat. no. A32959, ThermoFisher Scientific) and 1 mM phenylmethylsulfonyl fluoride (PMSF). The cells in the RIPA buffer were placed on ice for 20 min, and were then sonicated in ice cold water for 5 min. The lysates were centrifuged at 4 °C, and their protein was quantified using a BCA kit (cat. no. 23225, ThermoFisher Scientific). Equal amounts of protein were separated by 10% SDS-PAGE, and were then transferred onto a nitrocellulose membrane (cat. no. NBA 083C001EA, Protran) using a wet transfer system (Bio-Rad) or semidry Transferblot Turbo Transfer System (Bio-Rad). After the transfer, the membranes were blocked for 30 min in 5% skimmed milk dissolved in 1× TBST, and then incubated with 1:2000 primary antibodies in 5% BSA in TBST at 4 °C overnight.

The primary antibodies against HER-2 (cat no. MA5-140579) and Hsp70 (cat. no. MA3-006) were purchased from ThermoFisher Scientific; C-Raf (cat. no. sc-133); the galectin-3 (cat. no. sc-20157), Hsp90 (cat. no. sc-69703), Cdc37 (cat. no. sc-13129) were obtained from Santa Cruz Biotechnology; the Akt (cat. no. 9272S), ERK1/2 (cat. no. 9102S), and pERK1/2 (cat. no. 9101S) were obtained from Cell Signaling Technology; the HIF-1α (cat. no. NB-100-134) was obtained from Novus Biologicals; and the β-actin (cat. no. A1978) was obtained from Sigma-Aldrich.

The next day, the membranes were washed three times in TBST for 10 min, and then incubated with secondary antibodies (1:5000) diluted in 5% skimmed milk in TBST for 1 h at RT. As secondary antibodies, we employed HRP-conjugated anti-mouse (cat. no. sc-2954, Santa Cruz Biotechnology) and anti-rabbit antibodies (cat. no. HAF008, R&D System). The protein bands were detected with a ChemiDoc MP instrument (Bio-Rad) after treatment with an ECL reagent (cat. no. 170-5061, BioRad). All of the antigens and molecular weights in kDa are labelled next to the blots on the figures. Uncropped Western blot data are attached as Figure S6.

### 4.9. Cytoplasmic and Nuclear Extracts

The trypsinized cells from 10 cm dishes were lysed in 100 µL cytoplasmic extraction buffer (CEB) containing 10 mM HEPES pH 7.5 (cat. no. EC-230-907-9, Fisher Bioreagents), 10 mM KCl, 1 mM EDTA (cat. no. 20302.293, VWR Chemicals), 1 mM DTT (cat. no. BP172-25, Fisher Bioreagents), 0.5% Nonidet P-40 (cat. no. M158, AMRESCO), 0.5 mM PMSF, and protease inhibitors. The cells were allowed to swell on ice for 15 to 20 min, with tapping every 5 min. After that, the cells were centrifuged at 12,000× *g* at 4 °C for 10 min. The supernatants were collected as s cytoplasmic extract, and the cell pellets were washed the times with CEB. The remaining cell pellets were resuspended in 100 µL nuclear extract buffer (NEB) containing 20 mM HEPES pH 7.5, 400 mM NaCl, 1 mM EDTA, 1 mM DTT, 1 mM PMSF and protease inhibitors. After the resuspension, the cell pellets were placed on ice for 30 min, with vortexing every 5 min. The cell pellets were sonicated for 2 min and centrifuged at 12,000× *g* for 15 min at 4 °C. The supernatants were collected as a nuclear extract.

### 4.10. Tumorosphere Assay (Low Adhesion and Serum-Free 3D Spheroid Culture)

Per well, 2000 cells were seeded in flat bottom 96-well suspension culture plates (cat. no. 655185, Cellstar, Greiner Bio-One) in 100 µL DMEM or RPMI media containing 1× B27 (cat. no. 17504044, Gibco, Thermo Fisher Scientific), 25 ng/mL EGF (cat. no. E9644, Sigma-Aldrich), and 25 ng/mL FGF (cat. no. RP-8628, Thermo Fisher Scientific), 20% MethoCult H4100 (cat. no. 04100, STEMCELL Technologies). After 2 days, 50 µL media containing 0.3% DMSO or 3× the concentration of the indicated compounds were added to the wells in a 1:2 dilution-series, starting at 100 µM. After 72 h of treatment, 10% (*v*/*v*) alamarBlue reagent (cat. no. DAL1100, Thermo Fisher Scientific) was added to each well. Then, the fluorescence intensity (excitation 560 nm and emission 590 nm) was measured after 4 h using a H1 Hybrid Multi-Mode reader (BioTek).

### 4.11. Cell Viability Assay (2D Cell Proliferation)

Per well, 500–700 cells were seeded in 96-well flat bottom culture plates in 100 µL. After 24 h, the cells were treated with 0.1% DMSO vehicle control or test compounds diluted in a medium using a 1:2 dilution-series, starting at 100 µM. The plates were further incubated for 72 h. The cell viability was measured after the addition of 10% alamarBlue reagent per well (cat. no. DAL1100, Thermo Fisher Scientific). The fluorescence intensity (excitation 560 nm and emission 590 nm) was measured after 4 h by the use of a H1 Hybrid Multi-Mode reader.

### 4.12. Chick Chorioallantoic Membrane (CAM) Assay

The fertilized eggs were incubated at 37 °C in a 55% to 60% humidified egg hatcher incubator (MG100, Fiem). After 8 days, 2 million MDA-MB-231 cells were resuspended in 1× PBS and Matrigel (BD Biosciences) at a ratio of 1:1, and were then deposited within sterilized 7 mm diameter plastic rings cut from a plastic pipette (cat. no. LW4111, Alpha Laboratories Limited) on a surface of chicken embryo CAM. The xenografted tumors were treated with 20 µL 0.1% DMSO vehicle control or the test compound diluted in 1× PBS once a day, starting from 24 h after cell seeding. The microtumors were harvested after 5 days of treatment. The microtumors were collected in pre-weighted 1.5 mL Eppendorf tubes containing 500 µL PBS, and the tumor mass was determined by the difference of the weights.

### 4.13. Biomarker Analysis in the TCGA PanCan Atlas Database

The KRAS mutation status, and the normalized expression levels of *HIF1A* and *LGALS3* in log2 scale were obtained for the TCGA Pan Cancer cohort from the Xena browser [82]. In the KRAS mut analysis, the patients with a functional KRAS mutation status (i.e., having a deleterious or missense mutation) were considered for further analyses (*N* = 662), while the KRAS wt analysis comprised subjects without a functional KRAS mutation status (*N* = 8127). The patient samples were categorized into ‘low’ or ‘high’ expression groups based on the 20th and 80th percentiles of the expression levels for each gene, respectively. In the KRAS mut dataset, *LGALS3* cut-offs were defined at <12.06 for low and >13.9 for high, and for *HIF1A* at <10.9 for low and >12.45 for high. In the KRAS wt dataset, the LGALS3 cut-offs were defined at <10.33 for low and >12.19 for high, and for HIF1A at <10.89 for low and >12.54 for high. The number of samples from each tumor type, both in the high and low category, were plotted in order to assess the difference in enrichment between the groups.

### 4.14. Survival Analysis in the Pan-Cancer Patient Tumor Cohorts

A Kaplan–Meier survival analysis and a univariate Cox proportional hazard test were used to assess the difference in the overall survival between any two pre-defined patient groups from the TCGA pan-cancer dataset. The sub-groups were defined based the expression levels of *HIF1A* and *LGALS3*, as described under Biomarker analysis section above.

### 4.15. ATARiS Gene Dependency Data

In order to generate the ATARiS sensitivity plots, Excel files for each gene of interest were downloaded from the publicly-available database of the project DRIVE [47] (Drive Data Portal. Available online: https://oncologynibr.shinyapps.io/drive/, (accessed on 31 January 2020). The sensitivity score data were extracted in order to generate a double gradient heatmap plot using the GraphPad Prism software. A higher gene dependence in terms of 2D viability is indicated by an increasingly negative score, while 0 represents no or neutral effect.

### 4.16. Statistical Analysis

For the statistical analysis, the GraphPad Prism software (version 6) was used. We provide the sample size of independent biological repeats, *n*, for each data set in the figure legends. All of the graphs show mean values ± SEM across all of the technical and biological repeats. Unless otherwise stated, we employed one-way ANOVA with Tukey’s multiple comparison test or Student’s test to determine the statistical differences to the control samples. A *p*-value of <0.05 is considered statistically significant. The statistical significance levels are annotated in the plots as * *p* < 0.05; ** *p* < 0.01; *** *p* < 0.001; **** *p* < 0.0001.

## 5. Conclusions

We provided a novel mechanism for the way in which Hsp90 inhibition selectively affects K-Ras signaling. Our mechanism derived biomarkers (HIF-1α and galectin-3) suggest the evaluation of Hsp90 inhibitors in pancreatic cancer, and not just in kinase-addicted cancers. In addition, we identified six small molecule Hsp90/Cdc37 interface inhibitors with low micromolar activity. Currently, these compounds can serve as tools, and are starting points for the further development of such interface inhibitors. Importantly, the low on-target toxicity of such interface inhibitors makes them not only attractive for cancer therapy, but also as senolytics for life- and health-span extension.

## Figures and Tables

**Table 1:** Inhibition of the Hsp90/Cdc37 interaction measured in the split *Renilla* luciferase assays.

Compound	Hsp90/Cdc37 IC_50_ (µM)	N-Hsp90/Cdc37 IC_50_ (µM)
conglobatin A	23 ± 6	4.5 ± 0.8
×6506	5 ± 1	4.1 ± 0.4
×7648	18 ± 2	14 ± 4
×1127	41 ± 10	11 ± 1
×1540	44 ± 18	44 ± 9
×1742 (quercetin)	52 ± 6	42 ± 8
×1625	41 ± 8	94 ± 3

**Table 2:** Effect of compounds on 2D cell proliferation.

Cell Line (Tissue, KRAS Mutation)	×6506; IC_50_ (µM)	×1540; IC_50_ (µM)
SK-BR-3 (breast, n.a.)	36 ± 4	36 ± 3
MDA-MB-231 (breast, G13D)	32.6 ± 0.5	29 ± 3
MIA PaCa-2 (pancreas, G12D)	25 ± 2	23 ± 2
HCC-44 (lung, G12C)	28 ± 4	49 ± 6

**Table 3:** Effects of the compounds on 3D spheroid formation.

Cell Line	×6506 IC_50_ (µM)	×1540 IC_50_ (µM)
SK-BR-3 (breast, n.a.)	36 ± 4	64 ± 5
MDA-MB-231 (breast, G13D)	26 ± 1	58 ± 5
MIA PaCa-2 (pancreas, G12D)	30 ± 3	53 ± 6
HCC-44 (lung, G12C)	67 ± 5	52 ± 6

## Data Availability

The data presented in this study are all contained within this publication.

## Supplementary Materials

The following are available online at https://www.mdpi.com/2072-6694/13/4/927/s1, Figure S1: Split luciferase assay data and docking, Figure S2: Screening and new compounds in split luciferase assay, Figure S3: ATP-pocket assay and docking of new compounds, Figure S4: Survival and biomarker data with compound FRET-profiling, Figure S5: Additional spheroid data, Figure S6: uncropped western blot.
