# Supplementary material for: Novel Small Molecule Hsp90/Cdc37 Interface Inhibitors Indirectly Target K-Ras-Signaling
Source: Cancers (Basel). 2021 Feb 23;13(4):927. doi: 10.3390/cancers13040927 (PMC7927014; doi:10.3390/cancers13040927)

Some images on slide no 9, 10 and 16 are taken with selected image area. We can provide the Bio Rad Image Lab files for these blots

Figure 1G: Loading order: control, 2, 5, 10 uM conglobatin A, 17AAG

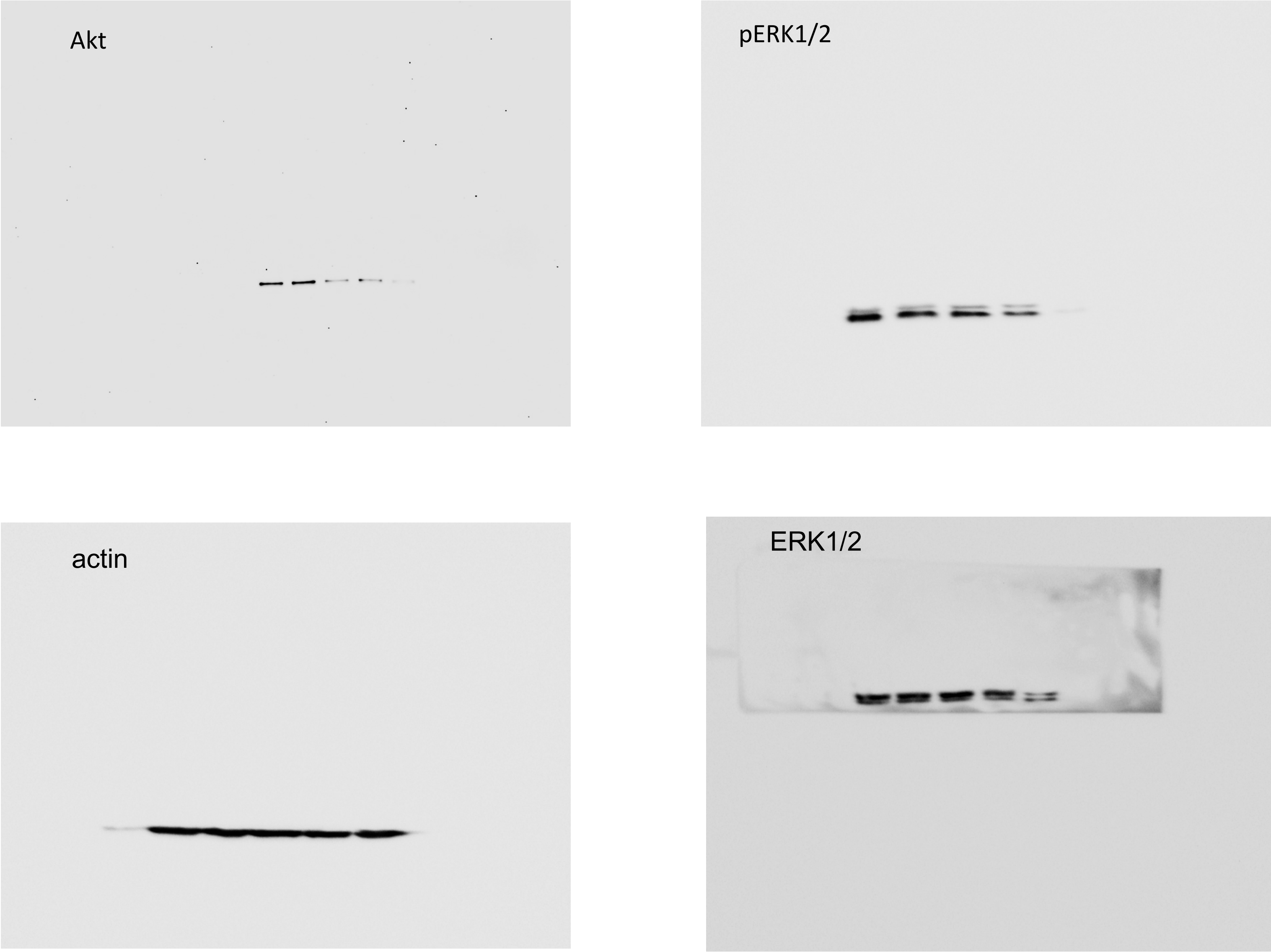

Figure 2A

Loading order for HEK293 + mGFPKRasG12V
Untransfected, control, 2, 5, 10 uM conglobatin A, 17AAG

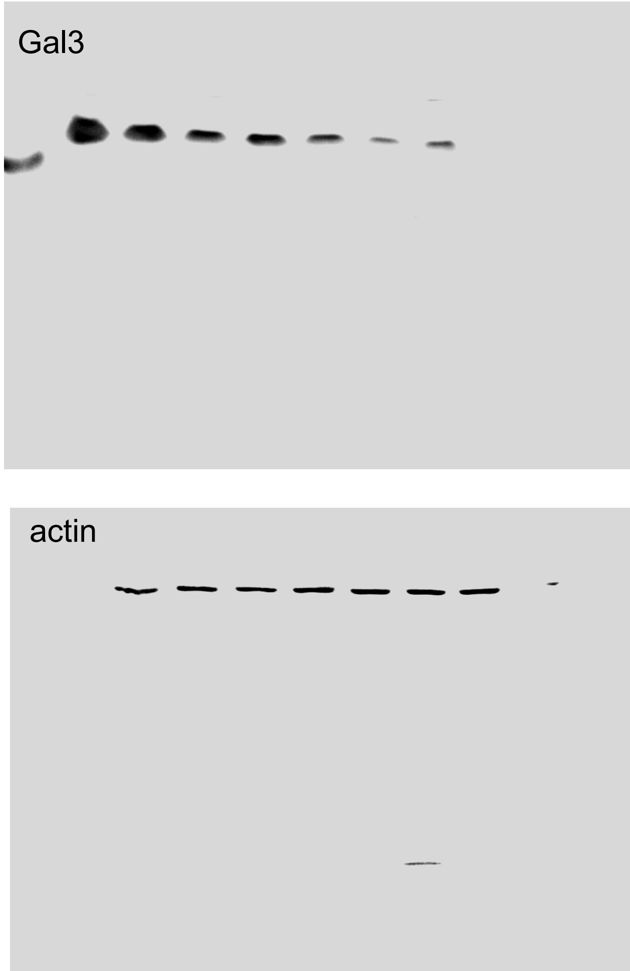

Loading order for MDA-MB-231
control, 2, 5, 10 uM conglobatin A, 17AAG

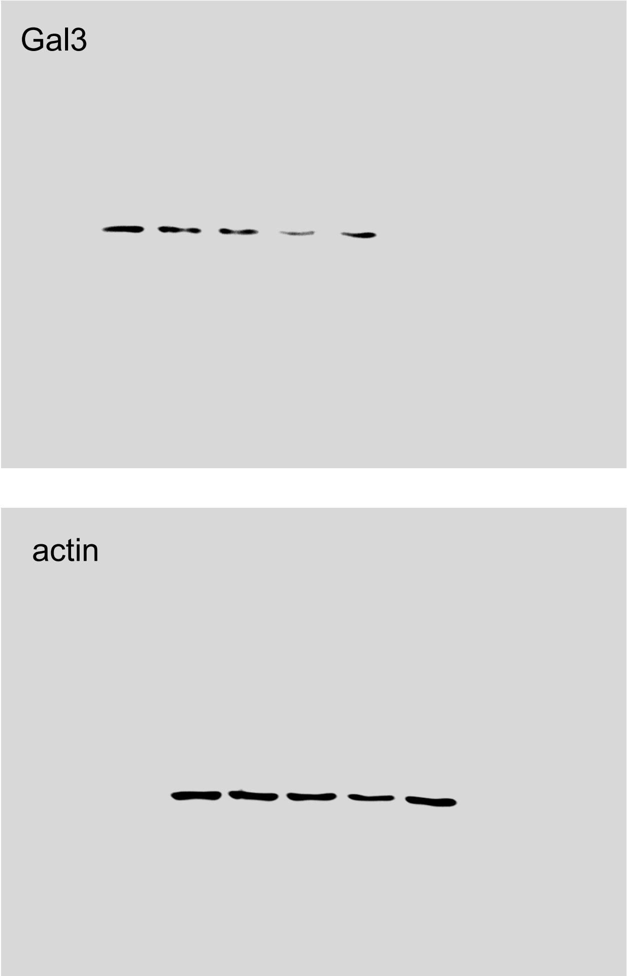

Figure 2B: Loading order as indicated in manuscript

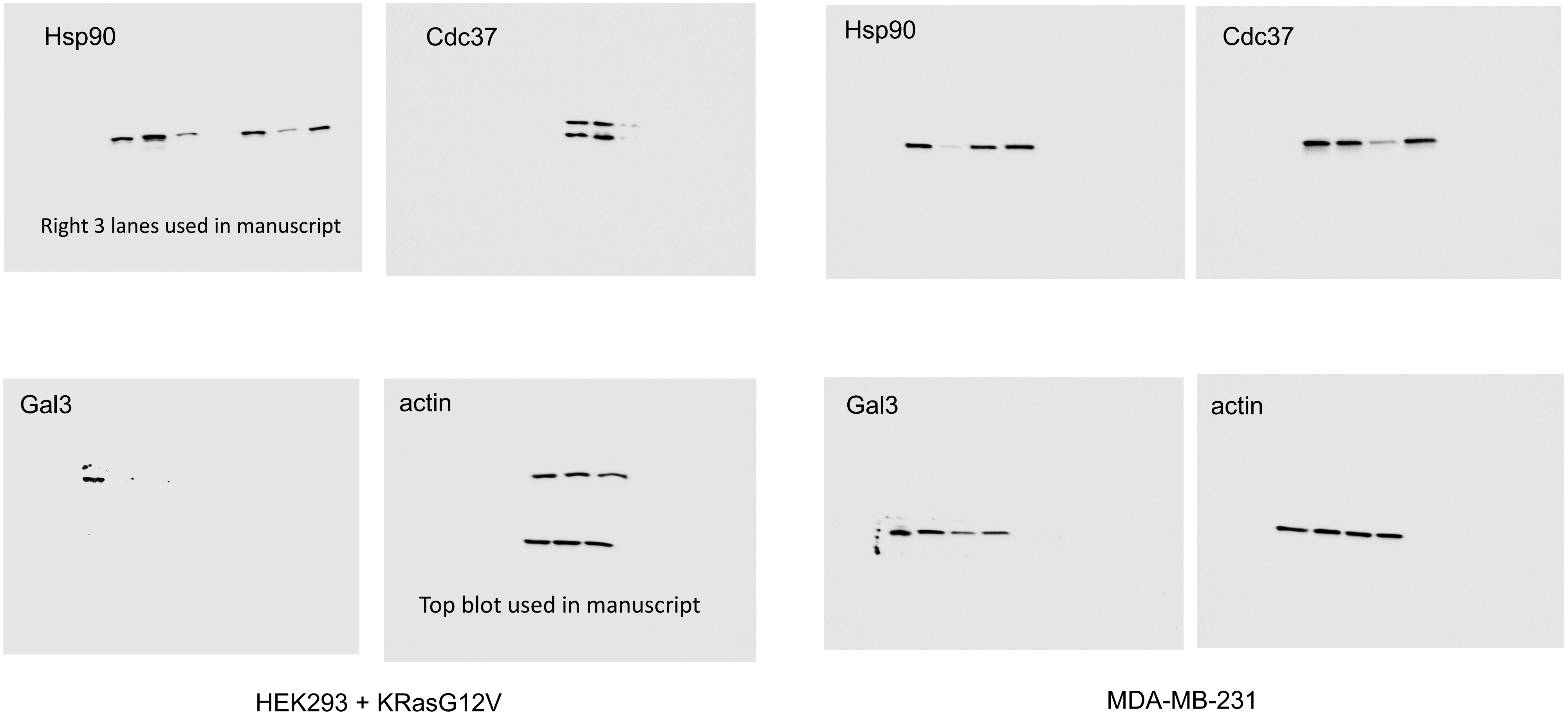

Figure 2D

Right 3 lanes: control, 100, 200 uM CoCl2

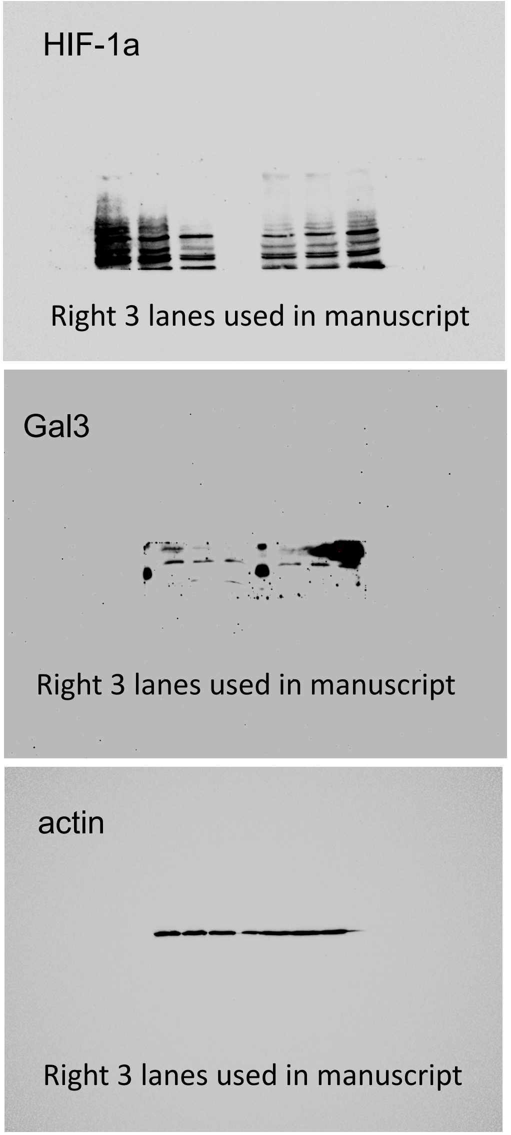

HEK293+KRasG12V

First: 3 lanes
control, 100, 200 uM CoCl2

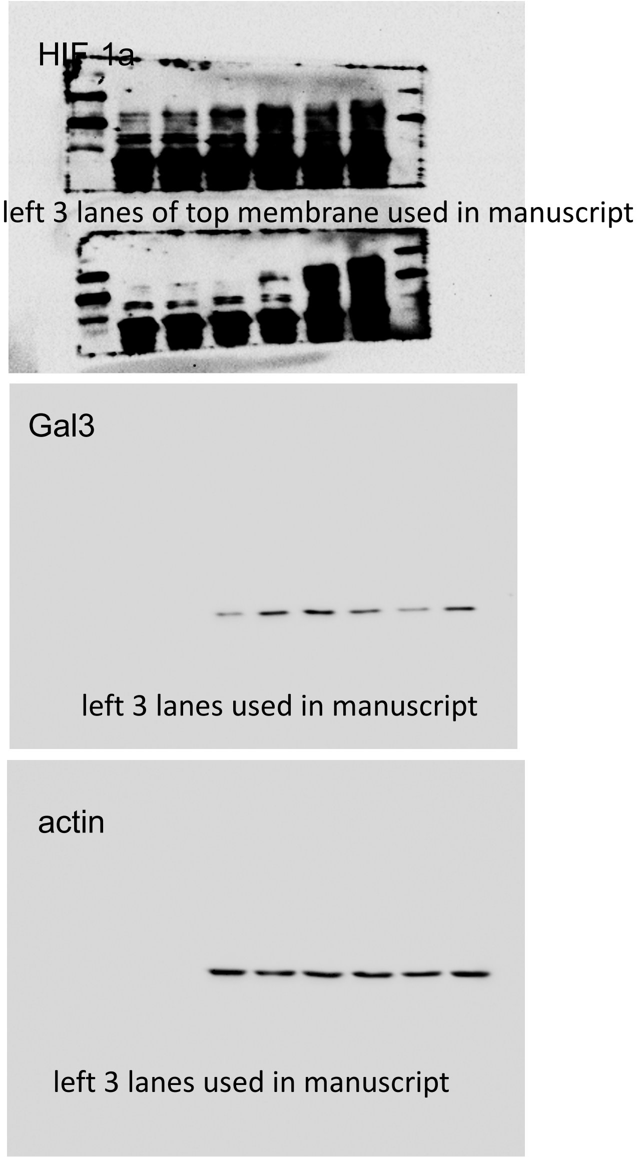

MDA-MB-231

Figure 2E: Loading order as indicated in manuscript

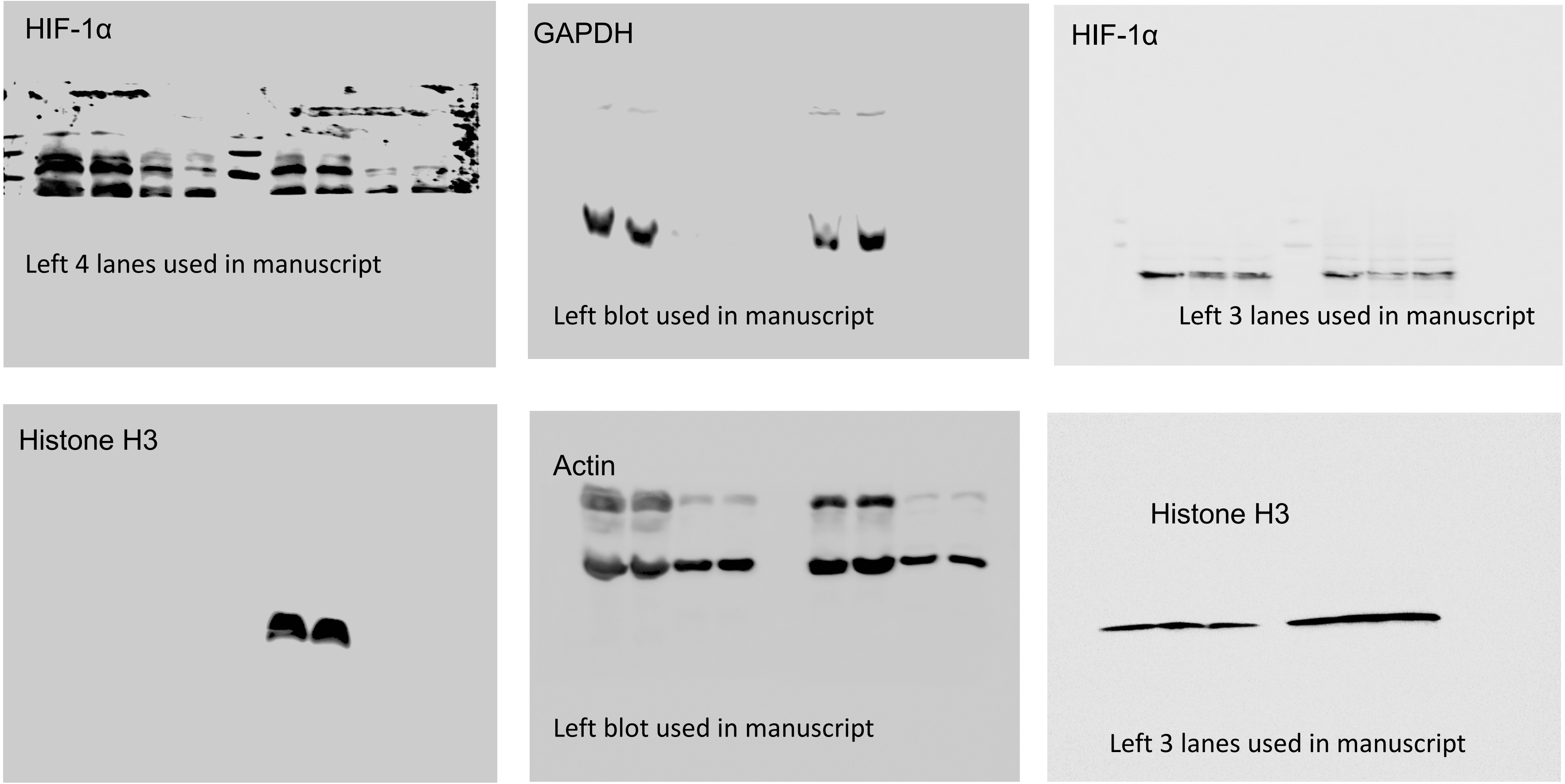

Figure: 2F: Loading order as indicated in manuscript

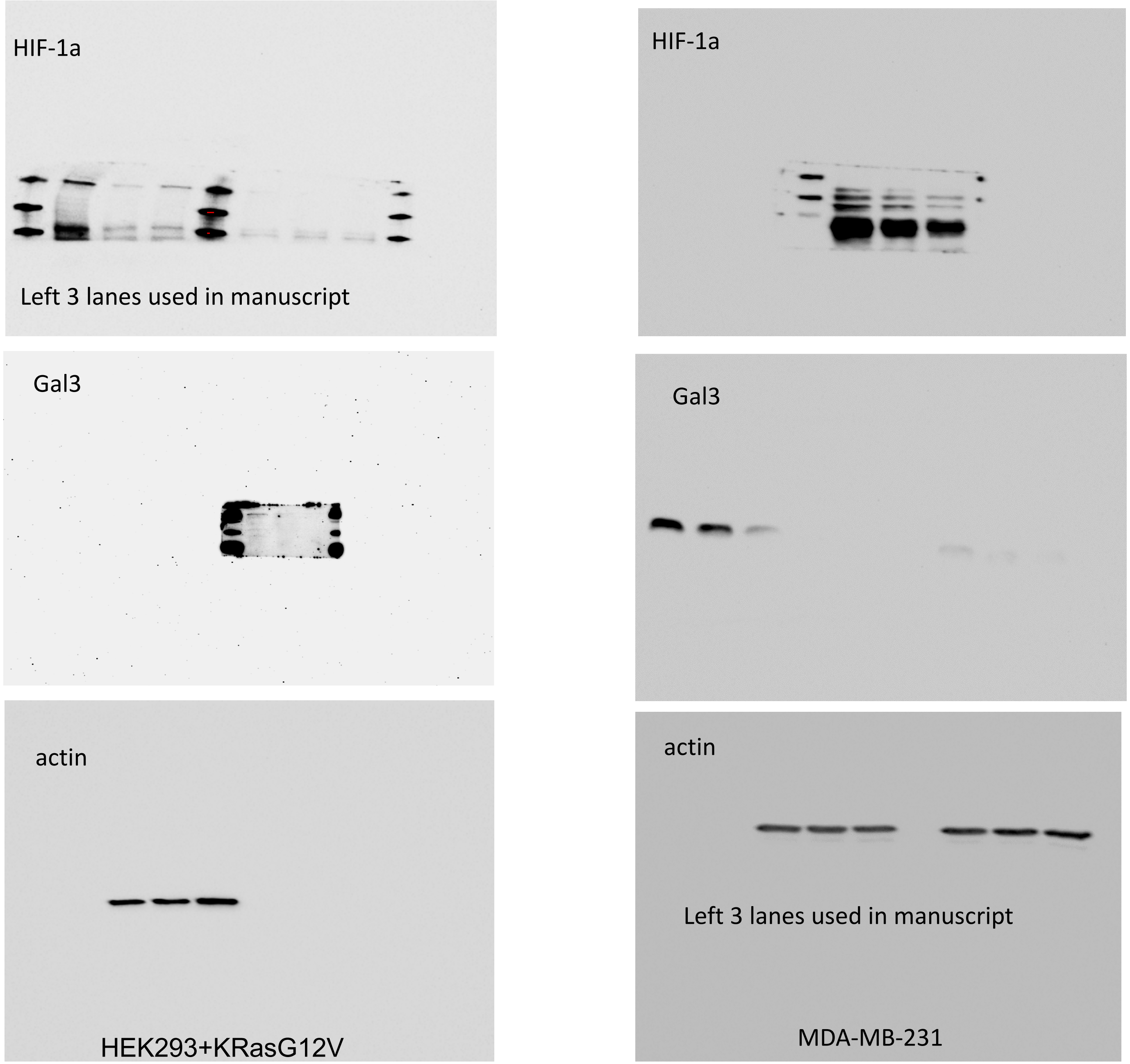

Figure 2G: Loading order as indicated in manuscript

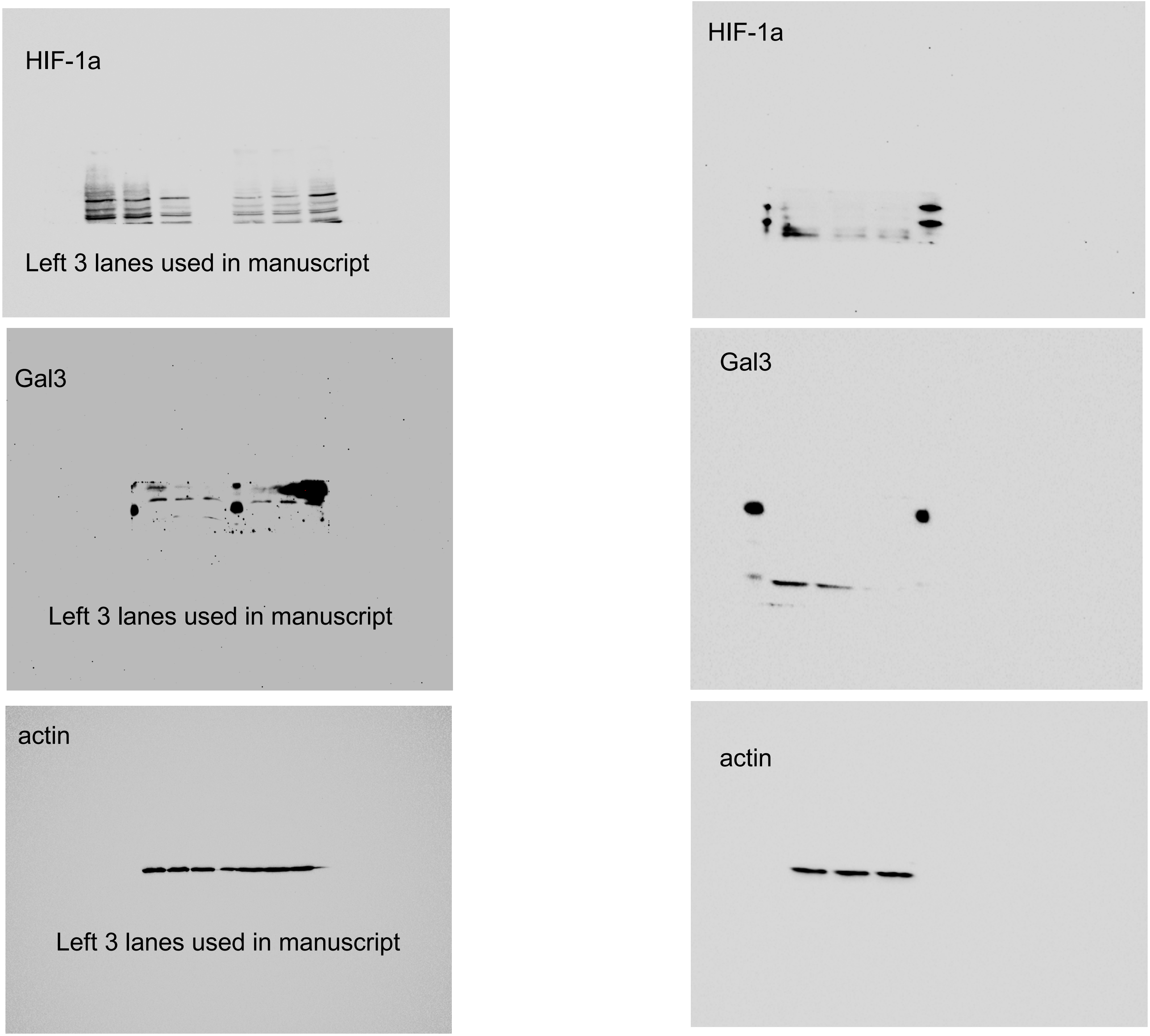

Figure 4A: SK-BR-3-x6506: : loading order: control 5, 10, 20 uM x6506, conglobatin A, 17AAG

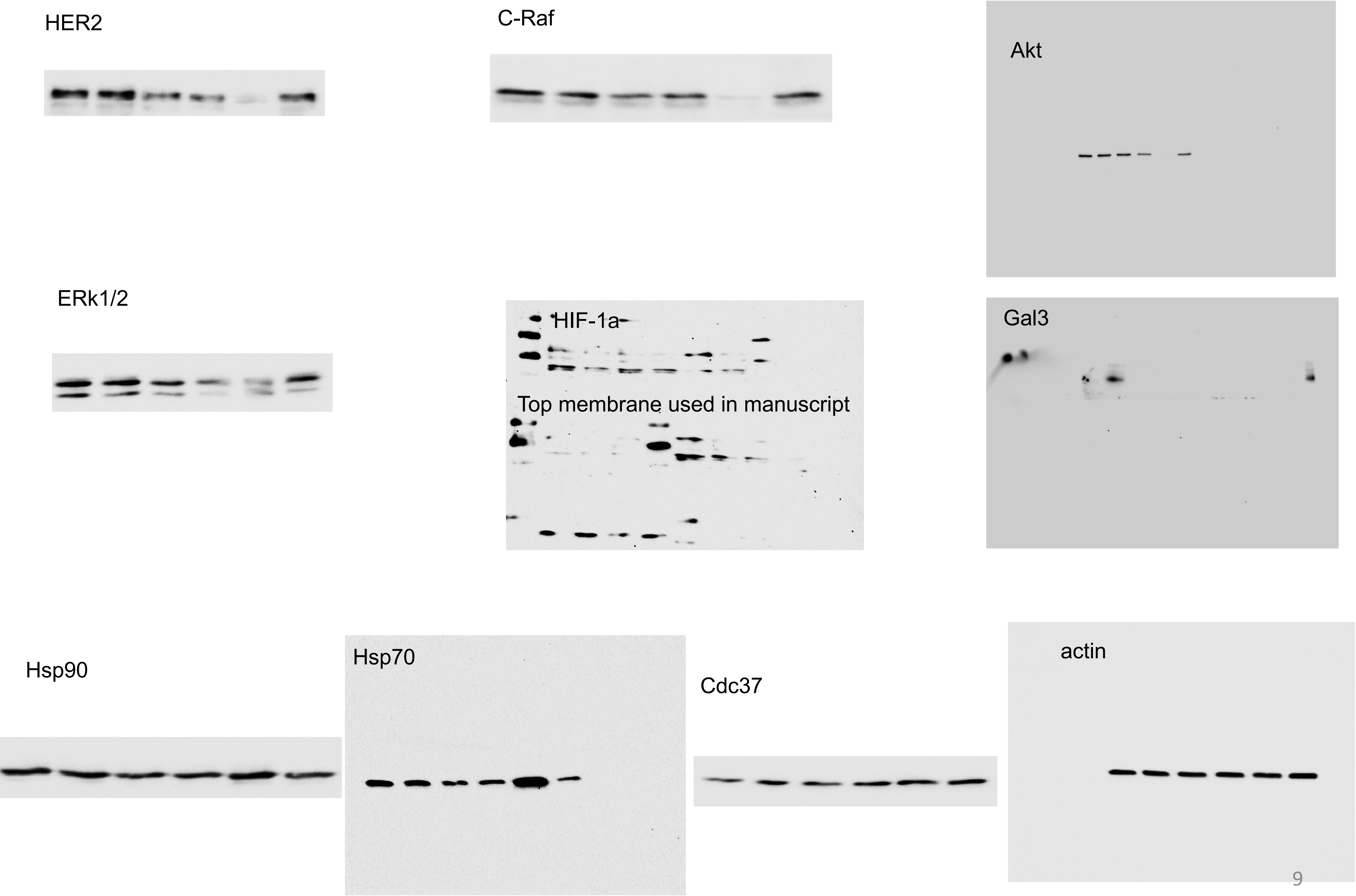

Figure 4A: SK-BR-3-x1540: loading order: control, 5, 10, 20 uM x1540, 17AAG

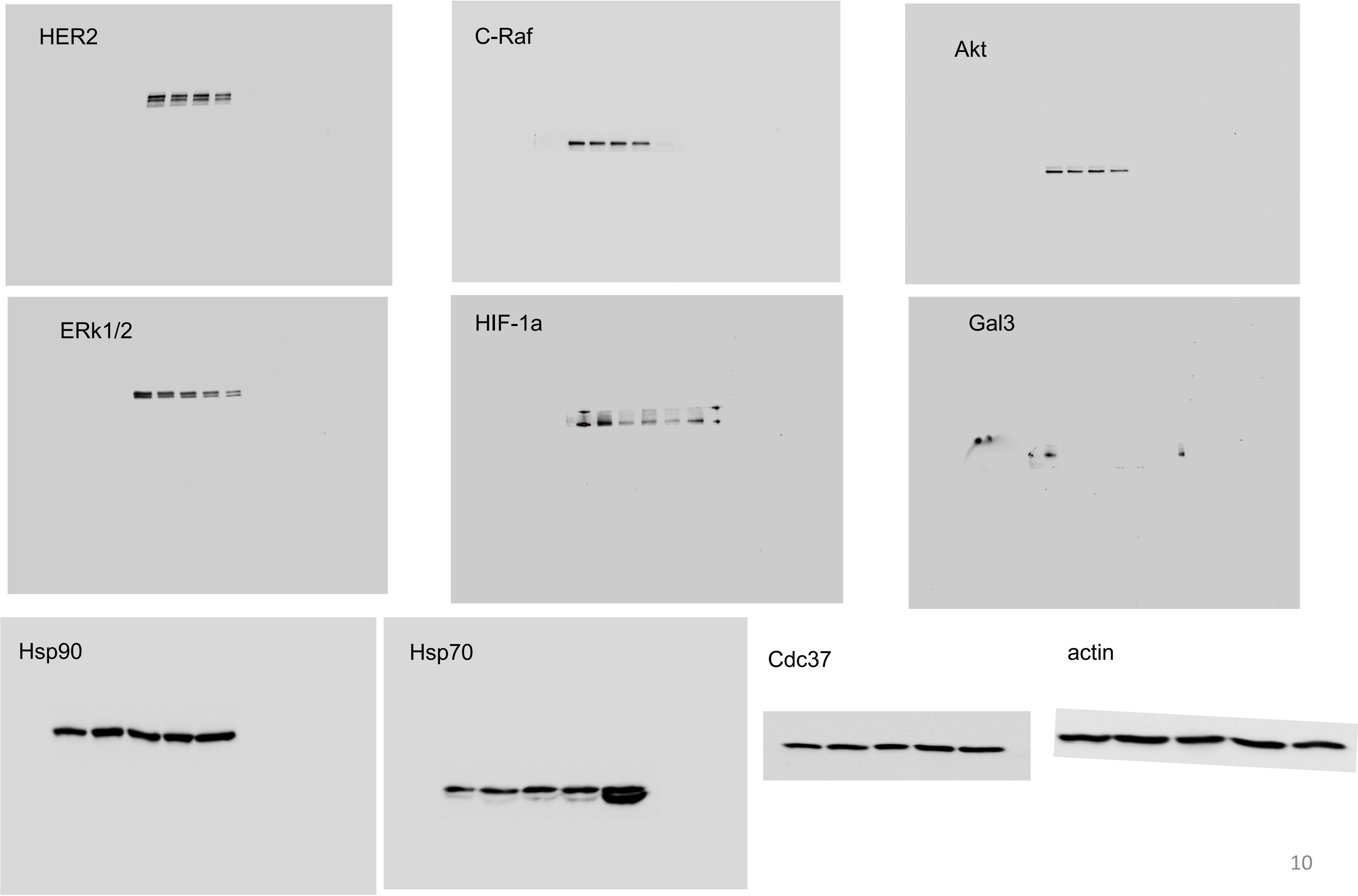

Figure 4B-MDA-MB-231-x6506: loading order: control 5, 10, 20 uM x6506, conglobatin A, 17AAG

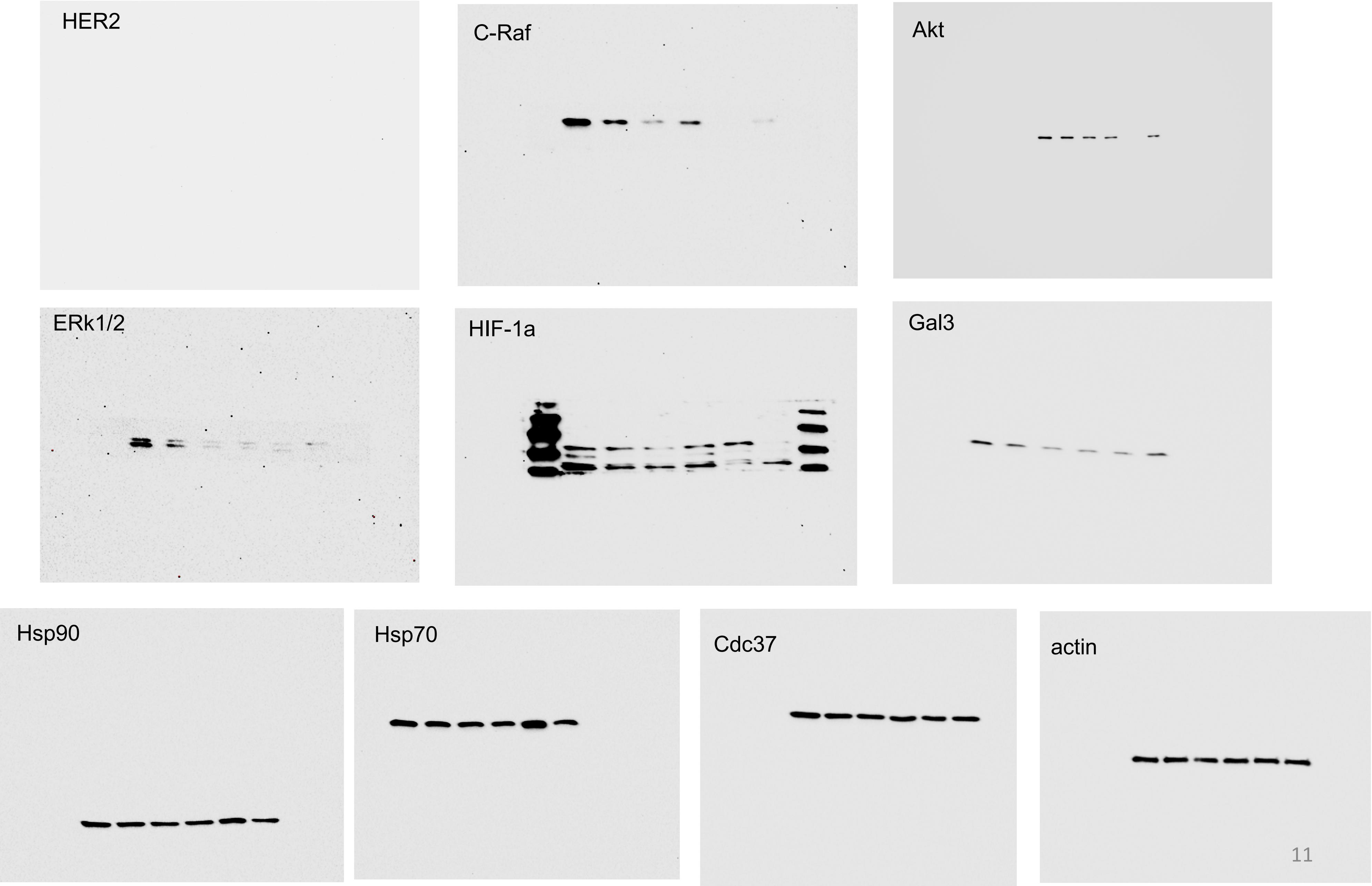

Figure 4B-MDA-MB-231-x1540: loading order: control, 5, 10, 20 uM x1540, 17AAG

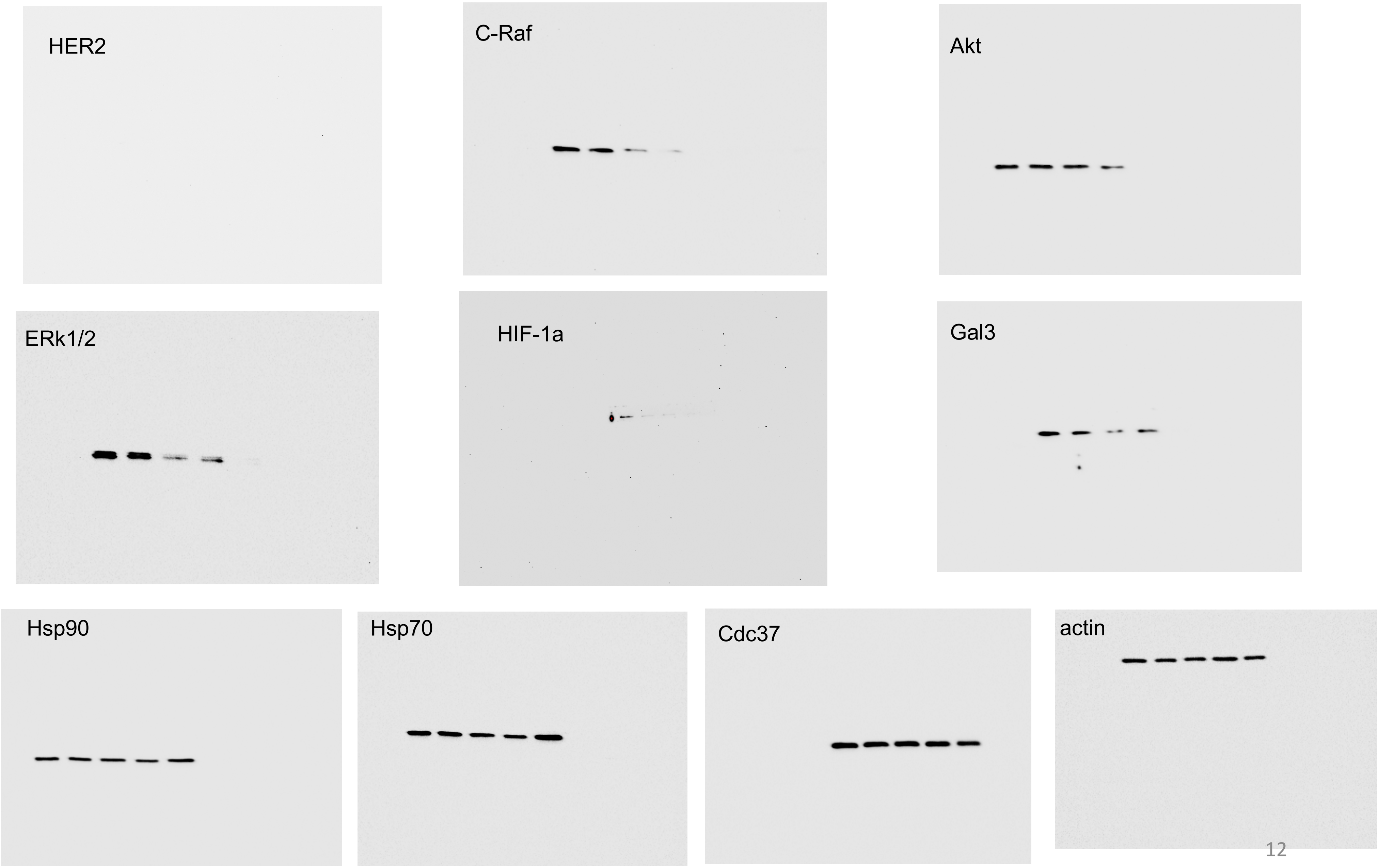

Figure 4C:MiaPaCa-2-x6506: : loading order: control 5, 10, 20 uM x6506, conglobatin A, 17AAG

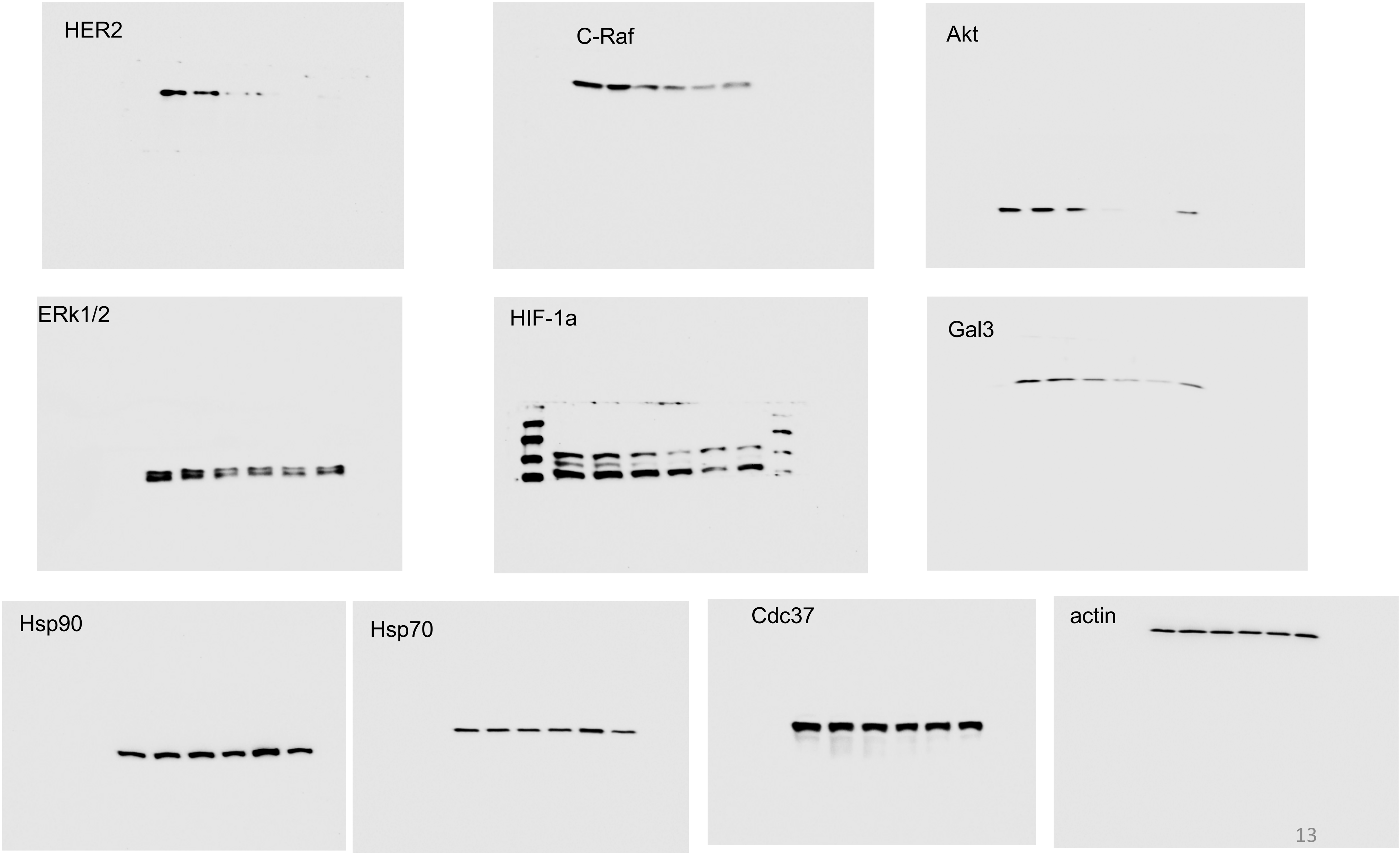

Figure 4C:MiaPaCa-2-x1540: loading order: control 5, 10, 20 uM x1540, 17AAG

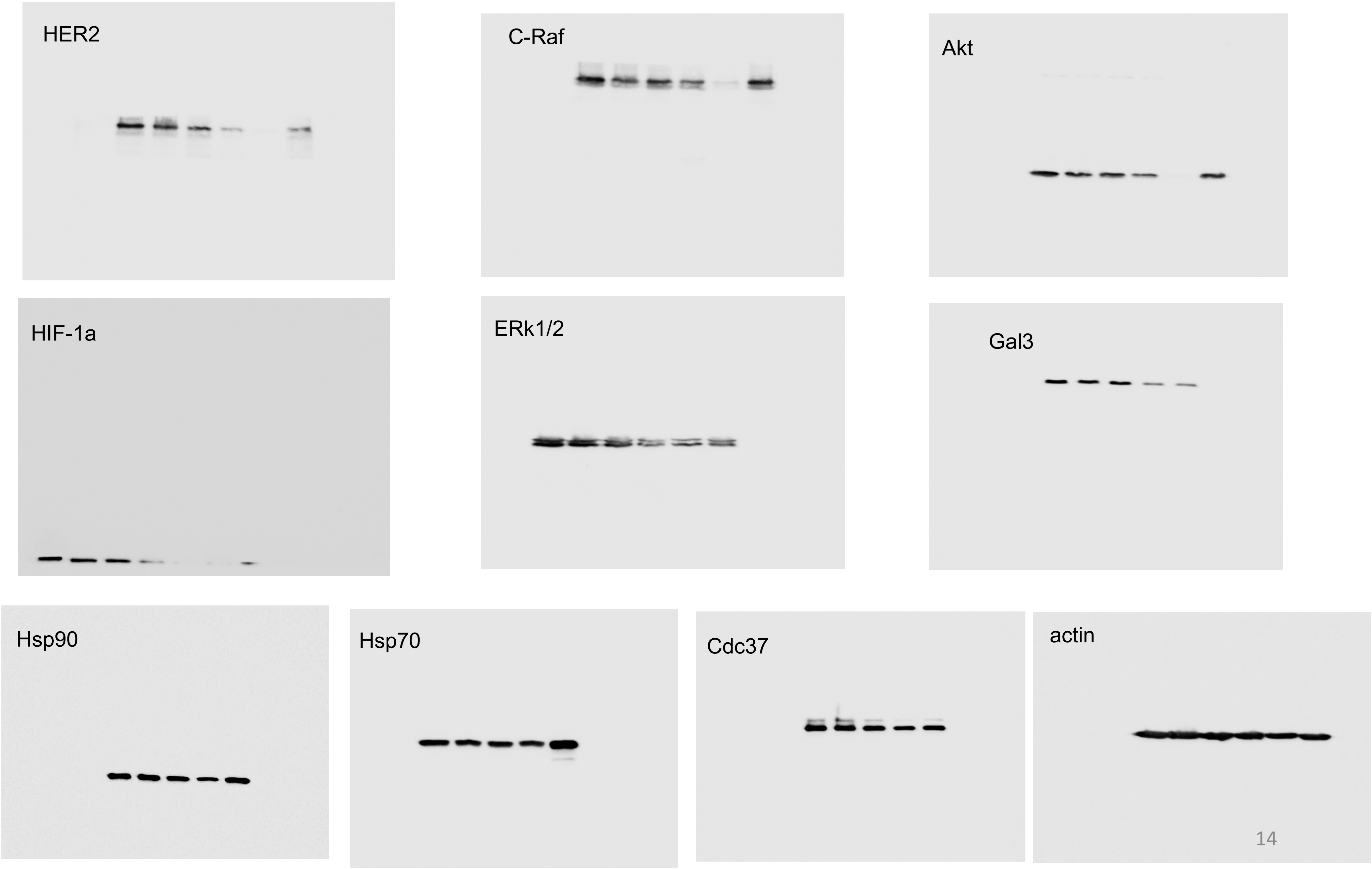

Figure 4D: HCC44-x6506: loading order: control 5, 10, 20 uM x6506, conglobatin A, 17AAG

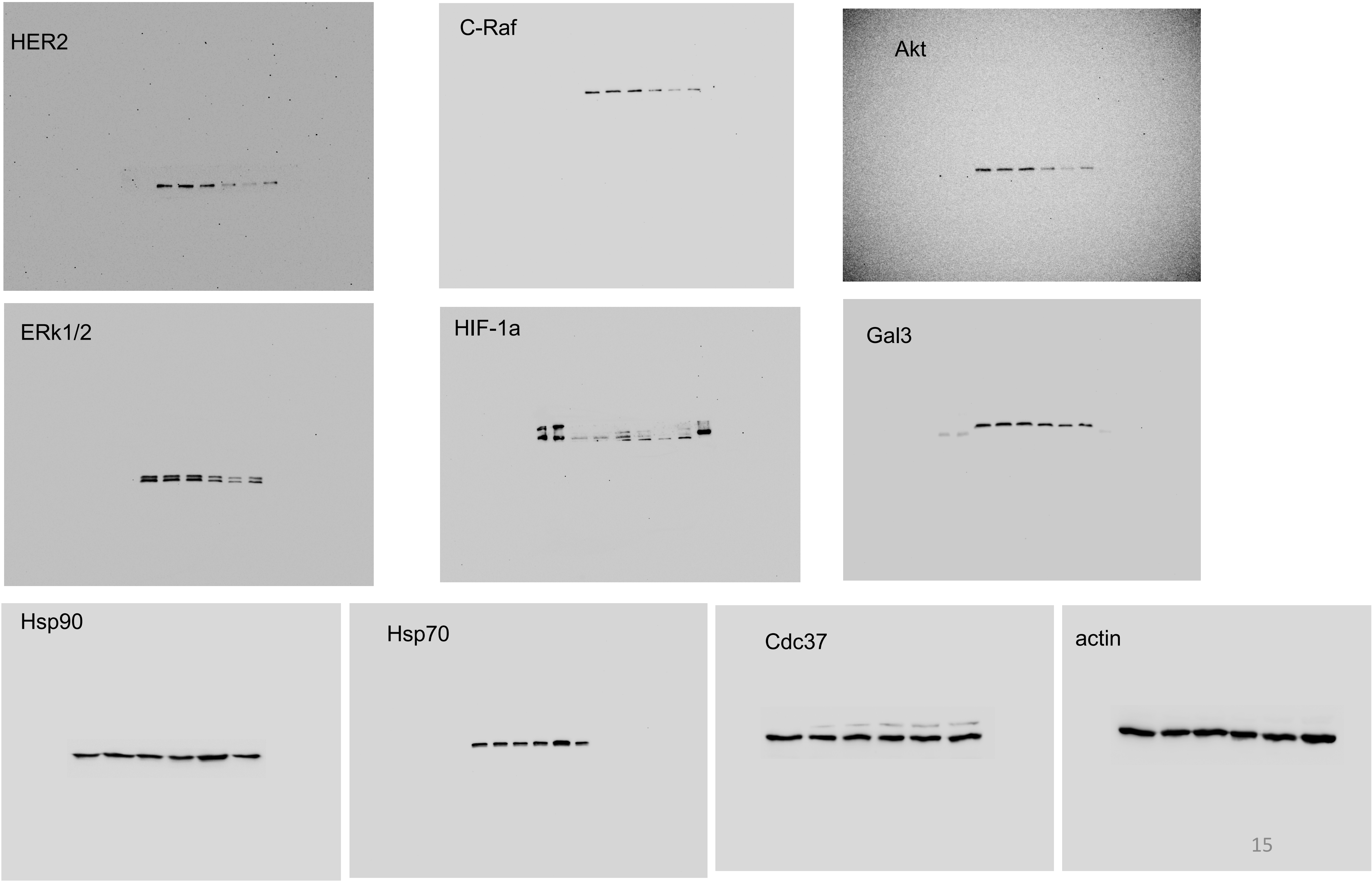

Figure 4D-HCC-44x1540: loading order: control, 5, 10, 20 uM x1540, 17AAG

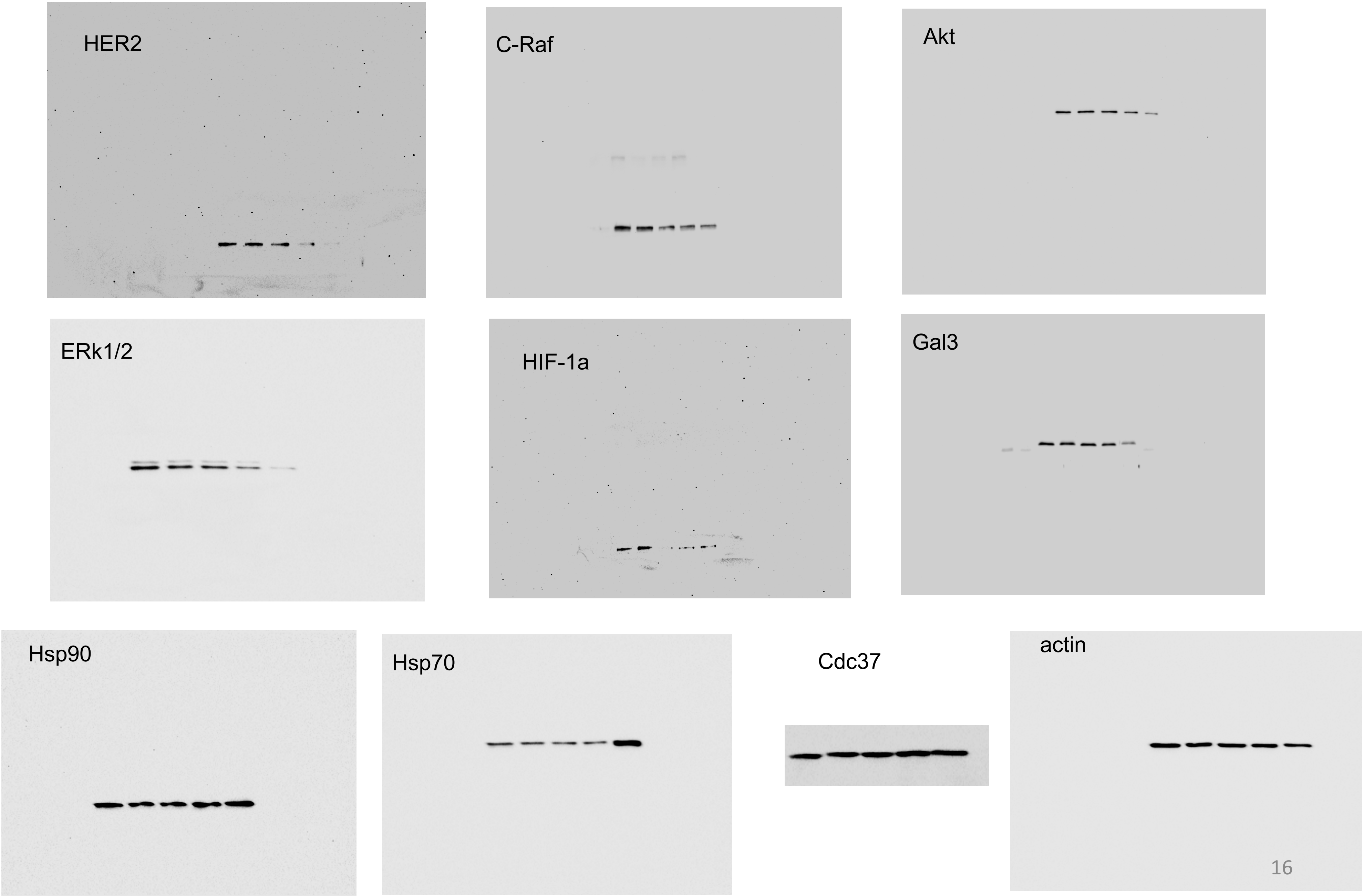

Supplement: Supplementary file 1 [file cancers-13-00927-s001.zip › cancers-1086784-Supplementary Materials/Figure S6 uncropped western blot.pdf]
