# Supplementary material for: Novel Small Molecule Hsp90/Cdc37 Interface Inhibitors Indirectly Target K-Ras-Signaling
Source: Cancers (Basel). 2021 Feb 23;13(4):927. doi: 10.3390/cancers13040927 (PMC7927014; doi:10.3390/cancers13040927)
Supplement: Supplementary file 1 [file cancers-13-00927-s001.zip › cancers-1086784-Supplementary Materials/Figures 1-5.pdf]

Supplementary Materials to Siddiqui et al.

Supplementary Figures

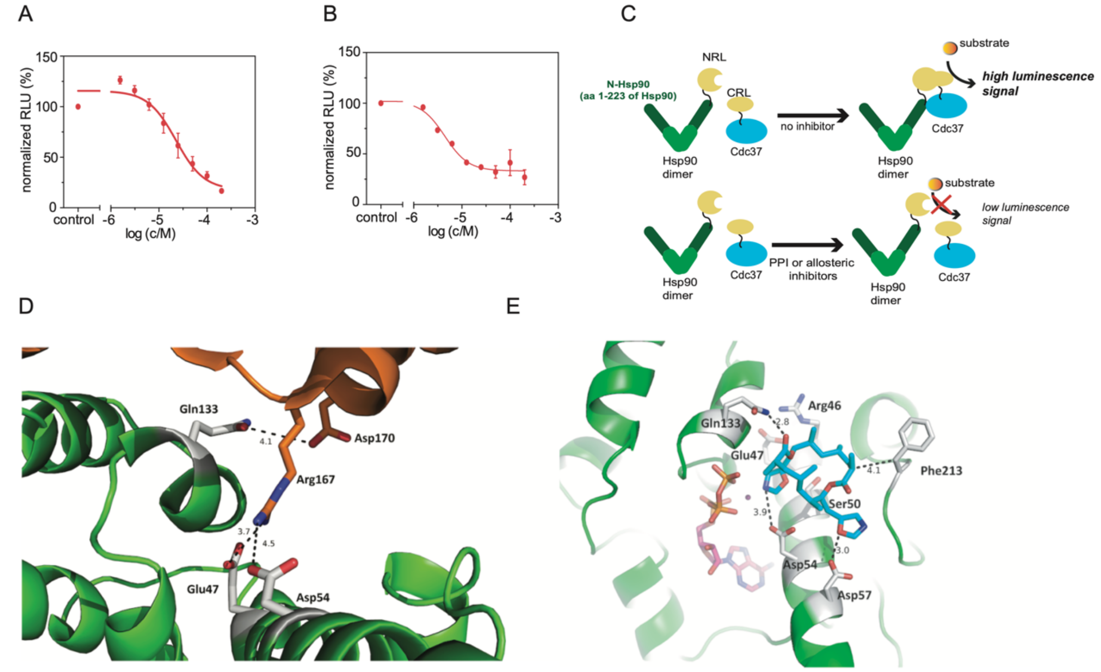

Figure S1: (A,B) Dose response data of conglobatin A tested in the split *Renilla* luciferase assay using the NRL-Hsp90/ Cdc37-CRL (A) or NRL-N-terminal-Hsp90 fragment/ Cdc37-CRL (B) reactions; n = 3 independent biological repeats. **(C)** Schematic representation of the split *Renilla* luciferase assay for the detection Hsp90/ Cdc37 complex inhibitors. In cell lysates the NRL-Hsp90 fragment interacts with the Cdc37-CRL fragment, which results in the formation of functional *Renilla* luciferase that is able to produce a high luminescence signal from the conversion of coelenterazine (top). In the presence of a Hsp90/ Cdc37 protein-protein interface inhibitor or allosteric inhibitor, NRL-Hsp90 (or NRL-N-Hsp90) is not able to interact with Cdc37-CRL, leading to a loss of signal (bottom). By using the NRL-N-Hsp90 fragment, Hsp90/ Cdc37 N-terminal protein-protein interface inhibitors can be distinguished from allosteric C-terminal inhibitors. **(D)** Molecular interactions between yeast N-Hsp90 (green) and the C-terminal domain of human Cdc37 (orange) in the crystal structure (PDB ID 1US7). The interacting residues in N-Hsp90 are shown as gray sticks. **(E)** N-Hsp90 (green; PDB ID 3T0Z) interactions with the

docked conglobatin A (cyan sticks). The interacting residues are shown as gray sticks, ATP as magenta sticks and magnesium as deep purple sphere. The interactions between the compound and Hsp90 are shown as dashed lines with distances in Ångströms.

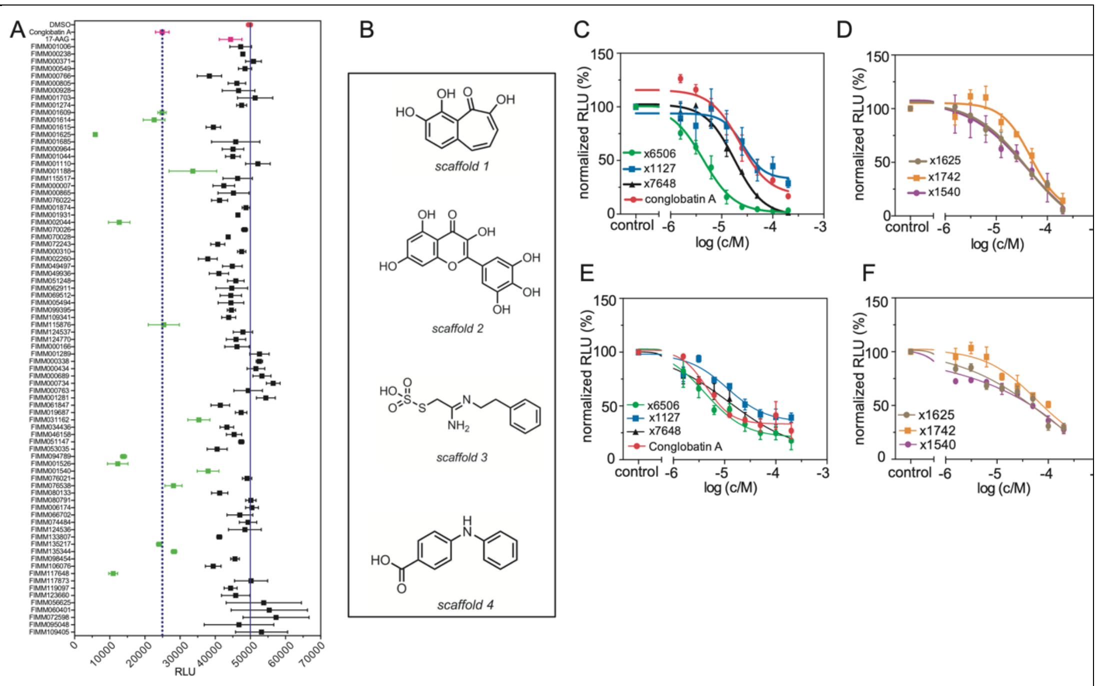

Figure S2: (A) Visualization of in silico hit validation data from FIMM screening 1 using the split *Renilla* luciferase assay. Compounds were tested at 20 µM concentration. Green dots represent hit compounds. **(B)** Structures of selected four scaffolds derived from FIMM screening 1. **(C,D)** Normalized dose response data of selected six drug-like hit compounds tested in the split *Renilla* luciferase assay using the NRL-Hsp90/ Cdc37-CRL reaction. Conglobatin A data are shown for comparison in (C); n = 3 independent biological repeats. **(E,F)** Dose response data of selected six drug-like hit compounds tested in the split *Renilla* luciferase assay using only the N-terminal fragment of Hsp90 in the NRL-N-Hsp90/ Cdc37-CRL reaction. Conglobatin A data are shown for comparison in (E); n = 3 independent biological repeats.

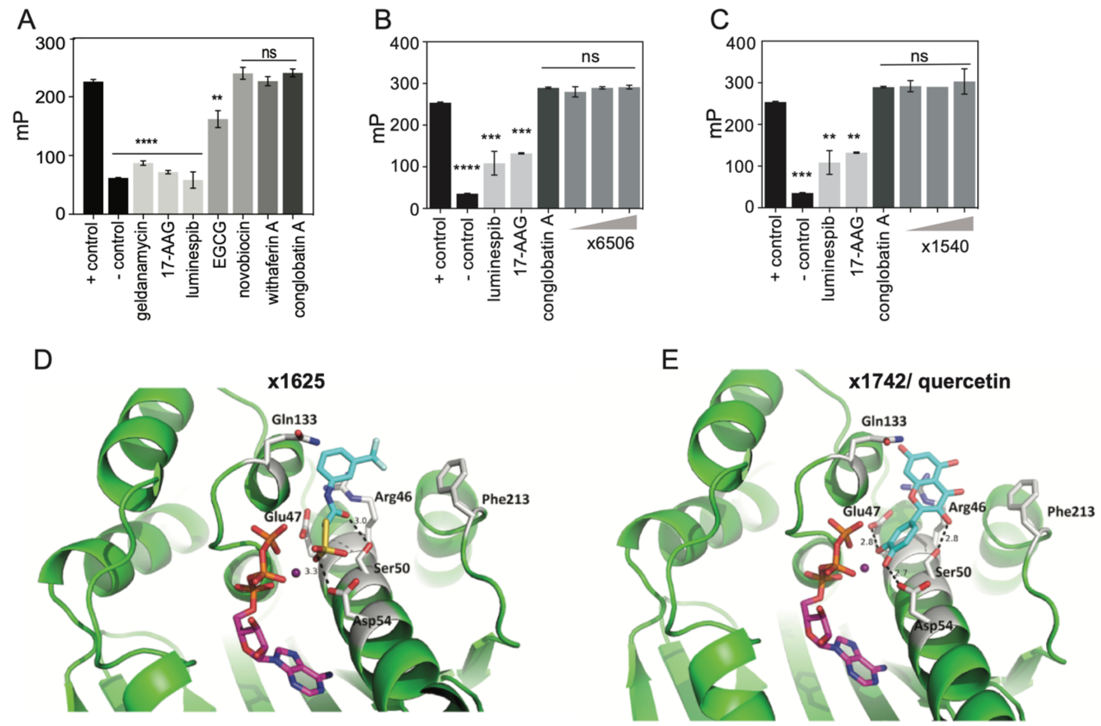

Figure S3: (A-C) Fluorescence polarization data from Hsp90 N-terminal ATP binding assay. The assay was validated using ATP-pocket inhibitors at 20 µM (geldanamycin, 17-AAG, luminespib), which significantly reduced the signal. Other inhibitors (20 µM) are classified as Hsp90/ Cdc37 interaction inhibitors (A). Testing of selected hit compounds x6506 (B) and x1540 (C) in the same assay at 12.5 µM, 25 µM or 50 µM. Concentrations of other inhibitors as in (A); n = 1 independent biological repeat. All samples were measured in duplicates. **(D,E)** N-Hsp90 (green; PDB ID 3T0Z) interactions with the docked x1625 (D) and x1742/quercetin (E). The interacting residues are shown as gray sticks, ATP as magenta sticks and magnesium as deep purple sphere. The interactions between the compounds and Hsp90 are shown as dashed lines with distances in Ångströms.

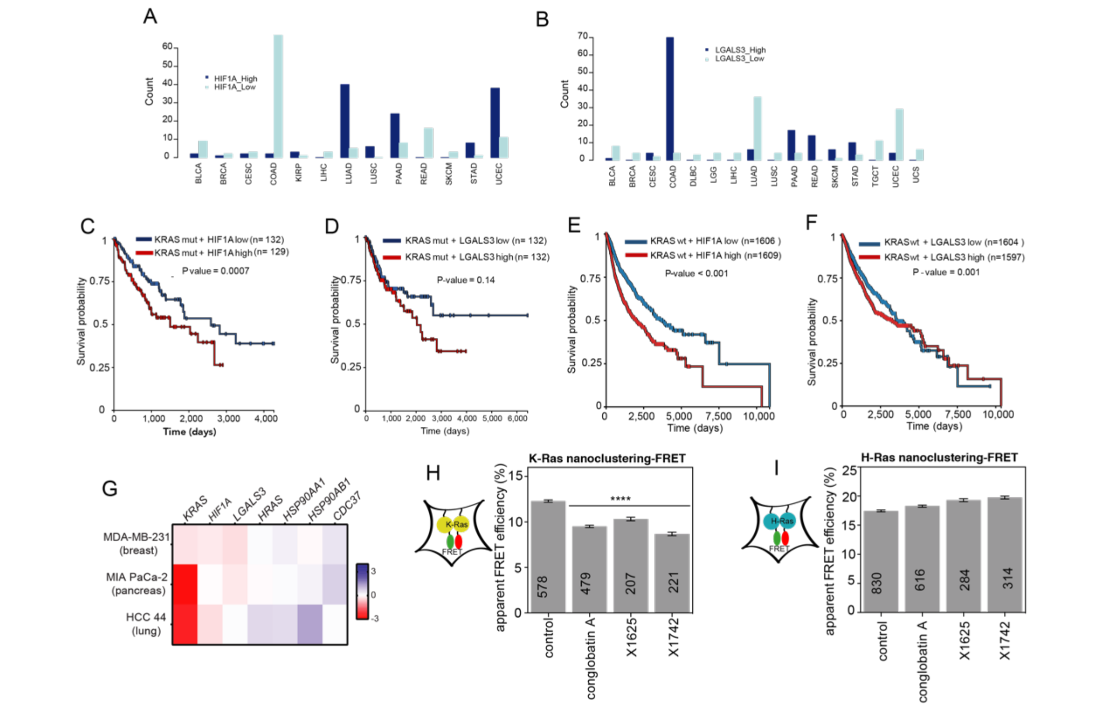

Figure S4: (A,B) The number of patients with a pathogenic KRAS mutation across various tumor types categorized by the expression levels of HIF-1α/ *HIF1A* (A) or galectin-3/ *LGALS3* (B). Data were obtained from the TCGA Pan cancer dataset and the cancer type abbreviations correspond to those employed therein. Note, several tumor types, including LUAD (lung adenocarcinoma) and PAAD (pancreatic adenocarcinoma), were enriched for samples having high HIF-1α expression, but only PAAD shows in addition an enrichment for samples with high expression of galectin-3 levels. **(C,D)** Patient survival data across all of the KRAS mutant samples in the TCGA Pan cancer dataset, categorized based on low (blue) or high (red) levels of HIF-1α/ *HIF1A* (C) or galectin-3/ *LGALS3* (D). **(E,F)** Patient survival data across all of the KRAS wild type samples in the TCGA Pan cancer dataset, categorized based on low (blue) or high (red) levels of HIF-1α/ *HIF1A* (E) or galectin-3/ *LGALS3* (F). **(G)** ATARiS sensitivity score heatmap of the indicated cell lines. Negative values (red) indicate sensitivity of the cell line proliferation to the knockdown of the shown genes. **(H,I)** K-RasG12V- (H) and H-RasG12V- (I) nanoclustering-FRET in HEK cells co-transfected with mGFP- and mCherry-tagged RasG12V. Cells were treated for 24 h with 0.1 % DMSO vehicle control, 2 μM conglobatin A or 5 μM of indicated hit compounds. The numbers in the bars indicate the number of analyzed cells; n = 3 independent biological repeats.

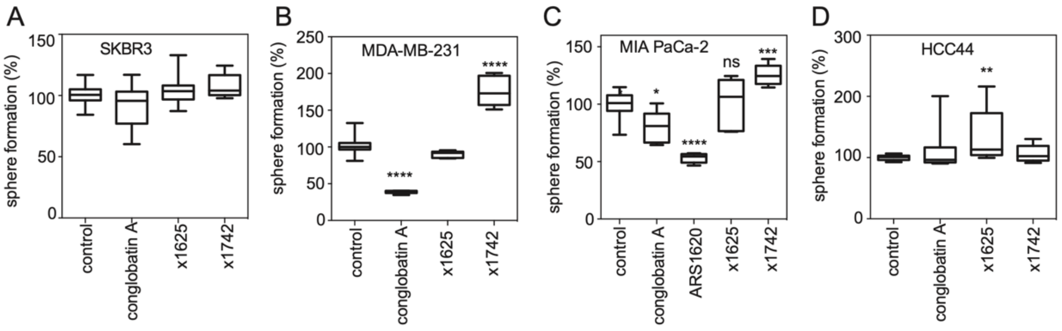

Figure S5: (A-D) Effect on indicated compounds after 72 h on 3D spheroid growth of several cancer cell lines cultured under low attachment and serum free conditions; n = 1 (C) or n = 2 (A,B,D) independent biological repeats. All samples were measured in hexaduplicates. Note the significant increase in sphere formation by x1742/ quercetin in the KRAS mutant breast cancer cell line MDA-MB-231 (B) and the pancreatic cancer cell line MIA PaCa-2 (C) that are likely to arise from off-target effects (i.e. off-target from Hsp90/ Cdc37) of this compound, which has several known activities and targets according to Drugbank.ca entry DB04216.
